# Female Employment Reduces Fertility in Rural Senegal

**DOI:** 10.1371/journal.pone.0122086

**Published:** 2015-03-27

**Authors:** Goedele Van den Broeck, Miet Maertens

**Affiliations:** Department of Earth and Environmental Sciences, KU Leuven, Leuven, Belgium; London School of Hygiene and Tropical Medicine, UNITED KINGDOM

## Abstract

Economic growth and modernization of society are generally associated with fertility rate decreases but which forces trigger this is unclear. In this paper we assess how fertility changes with increased labor market participation of women in rural Senegal. Evidence from high-income countries suggests that higher female employment rates lead to reduced fertility rates but evidence from developing countries at an early stage of demographic transition is largely absent. We concentrate on a rural area in northern Senegal where a recent boom in horticultural exports has been associated with a sudden increase in female off-farm employment. Using survey data we show that employed women have a significantly higher age at marriage and at first childbirth, and significantly fewer children. As causal identification strategy we use instrumental variable and difference-in-differences estimations, combined with propensity score matching. We find that female employment reduces the number of children per woman by 25%, and that this fertility-reducing effect is as large for poor as for non-poor women and larger for illiterate than for literate women. Results imply that female employment is a strong instrument for empowering rural women, reducing fertility rates and accelerating the demographic transition in poor countries. The effectiveness of family planning programs can increase if targeted to areas where female employment is increasing or to female employees directly because of a higher likelihood to reach women with low-fertility preferences. Our results show that changes in fertility preferences not necessarily result from a cultural evolution but can also be driven by sudden and individual changes in economic opportunities.

## Introduction

Developing countries have high fertility rates and face rapid population growth, leading to various environmental and social concerns [1]. In Sub-Saharan Africa (SSA) total fertility rates are among the highest in the world with 5.1 births per woman in 2012 compared to 1.7 for high-income countries [2]. A reduction in fertility rates is considered to be beneficial for these countries as it is associated with improved child and maternal health, empowerment of women, and poverty and hunger alleviation [3,4]—and hence contributes to achieve the first, third, fourth and fifth Millennium Development Goal (MDG).

Since the 1994 International Conference on Population and Development (ICPD), many developing countries have invested in family planning programs to reduce fertility rates. These programs mainly focus on improving knowledge about birth control and access to contraceptives. Total fertility rates (TFR) dropped since the 1990s but less so in SSA than in other low- and middle-income regions. In SSA the TFR decreased from 6.4 in 1990 to 5.1 in 2012, while in South-Asia TFR decreased from 4.2 to 2.6, and in Latin-America from 3.2 to 2.2 over the same period [2]. While improving the access to contraceptives is necessary for reducing fertility rates, it is not sufficient in countries where the demand for children remains high. For TFR to drop, fertility preferences need to change; either through a socio-cultural evolution or through socio-economic changes [5,6]. Economic growth and modernization of society are generally associated with TFR decreases—although there is some recent evidence that beyond certain levels of development, fertility rates increase again [7]—but it is not completely understood why and how [8–10].

In this paper we assess how fertility changes with increased labor market participation of women. Female employment affects fertility through three main channels: 1/ an income effect, 2/ a substitution effect, and 3/ an empowerment effect [11,12]. First, female employment contributes to total household income, and additional income can be invested in raising more children or in improving childcare quality. This income effect can lead to increased or reduced fertility, but generally fertility drops if income rises [13]. Second, employed women have a higher opportunity cost of raising children, and substitute productive labor for reproductive labor. This substitution effect results in decreased fertility. Third, working outside the household and earning an own income empowers women. If women have lower-fertility preferences than men—which has been documented to be the case for SSA [14]—women’s empowerment within the household will reduce fertility rates. Through employment women widen their social network, which can lower fertility preferences and increase knowledge about birth control [15].

The empirical relation between female employment and fertility has been documented for high-income countries, mainly through cross-country studies (e.g. [16,17]). Micro-economic studies and empirical evidence from developing countries, and especially from rural areas, are extremely scarce. Lower individual fertility due to employment of women has been documented for the Netherlands [18], United States of America [19], China [20] and urban areas in SSA [21,22]. Micro-economic evidence is needed to further elucidate whether female employment and fertility decreases are part of a cultural evolution or whether an economic revolution in female employment can trigger fertility decreases. Evidence from low-income countries is important because effects may differ in a setting of early and slow demographic transition, as in many countries in SSA [8,9]. Effects may differ because the cost of raising children is low, social security is largely absent, reproductive norms are different and female empowerment is low.

In this paper we analyze the effect of female employment on fertility in the Saint-Louis region in Senegal. This is a relevant case for two reasons. First, Senegal has a TFR of 5.0, which is one of the highest in the world [2]. The transition towards lower fertility started in Senegal, in the early 1970s in urban areas and the late 1980s in rural areas, but is particularly slow [23]. The Senegalese government is investing in family planning programs, especially targeting rural areas, with the aim of increasing the contraceptive prevalence rate to 27% by 2015 [24]. Our results can inform such policy to render programs more successful by directing them to regions where women have lower-fertility preferences. Second, female employment in the Saint-Louis region increased rapidly since 2005 as a result of a horticultural export boom. Increased investments in the horticultural export sector created employment opportunities for rural women while these women hardly participated in the labor market before the boom [25]. This represents an ideal case to study how fertility changes with increased female employment in a poor, rural area.

## Materials and Methods

### Ethics statement

We use data from a socioeconomic survey of rural households in Senegal. Households were chosen randomly from a list of households at the community level. The respondents were heads of households and their wives; no minors were interviewed. Respondents participated in the survey voluntarily, based on verbal consent. The purpose of the study was clearly explained to each respondent and based on that information respondents could choose to participate in the survey or not. When respondents gave their consent, their names and contact details were recorded by the enumerators. Written consent was not possible because a large share of the respondents are illiterate and not familiar with formal paperwork. The survey was carried out through face-to-face interviews in the local language. The interviews were implemented by trained enumerators. The authors did not implement any face-to-face interviews themselves but supervised the survey. The respondents provided information about all household members, including minors. No health related questions were asked. We carefully ascertained that respondents were not exposed to any risk or detriment during the survey. Data were analyzed in a strictly anonymous way and only used for research purposes. This work was done in accordance with the Helsinki Declaration. At the time the survey was designed and implemented, the Social and Societal Committee of KU Leuven did not yet exist and KU Leuven did not review socioeconomic survey research. Following completion of the study, we consulted Social and Societal Committee, who advised: “[the researchers] have informed the commission in detail about the research protocols and procedures that were involved in the study, and the commission concluded that these protocols are largely consistent with the standards of ethical research practices at our university.”

### Research area

Our research area covers three rural communities (Gandon, Fass and Diama) in the Saint-Louis region in the north of Senegal. This area was purposively chosen because it is one of the main horticultural export regions. A multinational holding invested in this area in 2003 and started to export cherry tomatoes in 2005. In the meantime the number of horticultural export companies in the region increased to five, and the cultivated area and product variety are still expanding. The five export companies are all located in the northern part of the area, north of Saint Louis town and the N2 road to Ross Bethio. Availability of land and water from the Senegal river are the main reasons for companies to establish in this area. In the southern area, south of Saint Louis town and the N2, no horticultural exports have been realized yet but one company already has a land lease deal in this area and is investing in irrigation infrastructure to start export activities from 2016 onwards. The companies produce vegetables on land leased from local rural communities, do post-harvest handling in their conditioning centers, and export produce to the EU; and rely on labor hired from the surrounding communities.

### Data collection

Survey data were collected in one round in April-June 2013. A stratified random sample of 500 households, clustered in 34 villages, was drawn, and a quantitative structured questionnaire was used. The survey provides household-level data on farm production, land and non-land assets, and living conditions, and individual-level data on demographic characteristics, employment history and off-farm earnings. Production and income data are collected for the 12 months period prior to the survey. Individual-level data are collected for all current household members (i.e. all persons who lived, slept and ate within the household compound during at least six months in the 12 months period prior to the survey), and children of the household head who already left the household. The sample of 500 households includes 1500 adult women above the age of 18; including wives, daughters and in-laws of the household head who live in the extended household. Data include the birth years for the surviving children of all these women; including children who do not live in the household anymore. This allows us to construct a detailed fertility history for these 1500 women. For the fertility analysis we only retain women in the age range from 18 to 40 because 18 is the lower age limit for formal wage employment and because we do not expect to see an impact for older women whose fertility decisions were already taken before the export boom. In addition, we suspect that the quality of the data on child birth years declines for older women. The final sample of women in the age cohort 18–40 includes 997 women of which 185 are employed outside the household and the household farm; the majority (66%) in horticultural export companies. While we only collected cross-sectional data in one survey round, we can construct a panel database for a limited number of variables as the survey includes detailed data on women’s fertility history and on entry into employment, and some other recall data. Additional data were collected from the sampled villages, on geographical and institutional characteristics, and from the five export companies, on production activities, sourcing strategies and working conditions. National export figures are from the FAOSTAT database.

### Employment and fertility calculations

**Fig 1:**
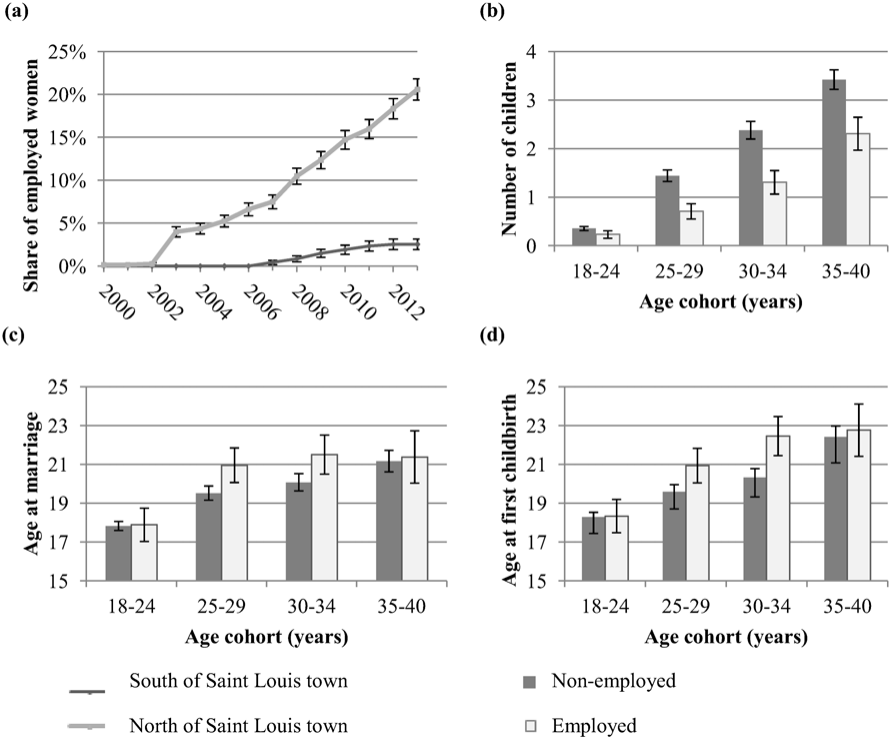
Female employment and fertility indicators. Female employment and fertility indicators calculated from survey data collected in 2013. (a) Evolution of the share of women employed in horticultural export companies over the period 2000–2013 in communities north and south of Saint Louis town (n = 1257). Figures include all women able to work (aged 18–65), and are based on recall questions about employment. (b) Average number of surviving children per woman for different age cohorts by employment status in 2013 (n = 997). (c) Average age at marriage for different age cohorts by employment status in 2013, conditional on being married or having been married (n = 997). (d) Average age at first childbirth for different age cohorts by employment status in 2013, conditional on having children (n = 997).

We distinguish a northern area, north of Saint-Louis where the export companies are located, and a southern area, south of Saint-Louis where no export companies are active yet. Female employment rates for these two areas (Fig. 1a) are calculated as the share of women (aged 18–65 and able to work) employed in horticultural export companies in a particular year in the period 2000–2012, based on recall questions about employment. Birth rates for the two areas are calculated as the total number of children born in a specific year divided by the total number of fertile women (aged 15–49) in that year. The TFR is calculated for the two areas as the sum of age-specific fertility rates for 5-year age cohorts (15–19, 20–24, up to 45–49) for the period 2007–2012. We compare fertility, age at first marriage and age at first child birth for employed and non-employed women in specific age-cohorts. We categorize the 997 women aged 18–40 in the sample in four age cohorts: 18–24, 25–29, 30–34 and 35–40. Age-specific fertility (Fig. 1b) is calculated as the average number of surviving children per woman for these age cohorts and for employed and non-employed women. We use the number of surviving children as fertility indicator rather than total number of live births, as is mostly done in economic studies [6]. We define employed women as women who participated in formal off-farm employment during the 12 months period prior to the survey (regardless of the length of that employment). Age at first marriage (Fig. 1c) and age at first childbirth (Fig. 1d) are calculated as average ages for the four age cohorts and for employed and non-employed women.

### Causal identification

We hypothesize that female employment reduces fertility but causal identification of this effect is difficult due to unobserved heterogeneity and reverse causality. Potential underestimation of the negative effect of female employment on fertility (or overestimation of the fertility-reducing effect of employment) is a particular concern as this may lead to wrong conclusions. Unobserved heterogeneity in social norms and in the initial empowerment of women may influence the likelihood of women to be employed and their fertility in opposite directions. If more empowered women or women in less traditional communities have a higher probability to be employed and a lower fertility, we may underestimate the negative effect of female employment on fertility (or overestimate the reduction in the number of children). Also reverse causality may lead to underestimated effects if women with (more) children have a lower likelihood to be employed [26]. Overestimation of the effect of female employment on fertility (or underestimation of the fertility-reducing effect) is less likely but could occur, e.g. if poorer women have more children and are more attracted to off-farm wage employment opportunities. We use difference-in-differences (DD) estimation, combined with matching, and Poisson regression models with village fixed effects and instrumental variables (IV) as strategy to reduce potential bias. We combine these methods in order to exploit the strengths of each of them and reduce potential bias to the extent possible and in order to check the robustness of the estimated effects. Randomized control trials (RCT) are sometimes put forward as the most credible causal identification strategy but an RCT in which employment is randomized across the female working-age population to measure the effects on fertility years later is not feasible and prone to a ‘faux exogeneity’ claim [27].

#### Difference-in-Differences estimation

We use difference-in-differences (DD) estimation to compare the number of surviving children per woman for employed/treated and non-employed/control women before and after the treatment. We use 2005, the start of the horticultural export boom when female employment was very low (Fig. 1a), as pre-treatment point and 2013, the year of the survey when female employment was very high (Fig. 1a), as post-treatment point. We use a balanced panel of the 997 women in the sample who were in the age category 18–40 in 2013. We use the fertility history data to derive the number of children a woman had in 2005, and recall data for other variable values in 2005. First, we estimate a simple DD model (Equation 1, DD) including a treatment variable (*T*_*i*_: a dummy variable for women being employed in 2013), a time variable (*t*: a dummy for the post-treatment year), and the interaction between these two (*T*_*i*_ *t*). Second, we estimate the DD model (Equation 2, DD with covariates) with a set of observable time-varying and time-constant pre-treatment characteristics *X*_*i*_. The vector *X*_*i*_ includes women’s age, literacy, marital status, religion and ethnicity, household land ownership and distance from the road in 2005. These variables might be correlated with female employment and fertility and are included to increase consistency and efficiency of the coefficient estimates. The coefficient *β*_*1*_ represents the time-invariant differences between the treated and control group and the coefficient *β*_*2*_ represents the effect of going from the pre-treatment year to the post-treatment year. Our main interest lies in the coefficient *β*_*3*_ that represents the DD estimator of the effect of female employment on fertility (*Y*_*i*_), as indicated in Equation 3.

$$Y_{i}=\beta_{0}+\beta_{1}T_{i}+\beta_{2}t+\beta_{3}T_{i}t+\varepsilon_{i}$$ (1)

$$Y_{i}=\beta_{0}+\beta_{1}T_{i}+\beta_{2}t+\beta_{3}T_{i}t+\beta X_{i}+\varepsilon_{i}$$ (2)

$$\beta_{3}=(Y_{T,t2}-Y_{T,t1})-(Y_{C,t2}-Y_{C,t1})\text{ with T treated and C control;}$$ (3)

Using the DD estimation, we are able to control for observable pre-treatment characteristics and for unobservable time-constant effects that might be correlated with both employment and fertility. However, the estimated effect of female employment on fertility might still be biased if employed/treated and unemployed/control observations are not similar in unobservable pre-treatment characteristics as treatment is not randomly assigned to women. We further control for heterogeneity in initial conditions by combining the DD estimation with Propensity Score Matching (PSM). We estimate a propensity score (PS) using the vector *X*_*i*_, match employed and unemployed women on the PS using Kernel matching, derive the matching weights, and use these weights in the DD estimation of the effect of employment on fertility within the common support region. Balancing properties are tested (S4 Table) and treated and control units are found to be similar in observable characteristics after matching. This approach resembles a quasi-experimental approach to create similarity in treated and control units through matching.

The DD estimation does not solve the issue of time-varying unobservable characteristics that are potentially correlated with female employment and fertility. An additional drawback is that we can only include a limited number of variables in the DD estimation for which recall information is available. Therefore, we combine this method with a cross-sectional Poisson regression that allows us to include more observable characteristics and to use instrumental variables to reduce bias from reverse causality and unobserved heterogeneity.

#### Poisson regression

We estimate the effect of female employment on fertility using cross-sectional regression analysis. As the number of children is a count variable, we assume a Poisson distribution of the dependent variable *Y*_*i*_ with the mean *μ*_*i*_ an exponential function of a vector of covariates *X*_*i*_. This vector includes our main variable of interest, a dummy variable for female employment, and a large set of control variables at individual level (women’s age, literacy, marital status, religion, ethnicity, and relation to the household head), household level (land ownership, livestock ownership, poverty status, and age, gender and literacy of the household head), and village level (distance from the road, presence of a female organization, and ethnic composition). Poverty status is calculated according to the Multidimensional Poverty Index (MPI) guidelines by the United Nations Development Program [28]. Households are considered to be poor if their MPI is higher than 0.33. These control variables capture observable characteristics that are likely correlated with female employment and/or fertility. Additionally, we include the number of children a woman had in 2005 to control for fertility preferences before the horticultural export boom and associated female employment started.

$$Pr(Y_{i}=y)=\frac{\left(e^{\left(-\mu_{i}\right)}\mu_{i}^{y}\right)}{y!};\mu_{i}=e^{\left(\beta X_{i}+u_{i}\right)},for\;y=0,1,2,\ldots ,$$ (4)

First, we estimate a standard Poisson regression with robust standard errors to correct for over-dispersion (Poisson). Second, we additionally include village fixed effects (Poisson Village FE). If unobserved norms and attitudes towards female employment and fertility are community-specific, village fixed effects may control for some part of the unobserved heterogeneity. Third, we apply a two-stage residual inclusion (2SRI) or control-function approach to further reduce the unobserved heterogeneity bias. A conventional two-stage least squares (2SLS) approach would lead to inconsistent estimates, because of the non-linearity of the Poisson model [29]. The distance to the nearest horticultural export company is used as an instrument. This is a relevant instrument as it has a large negative and significant effect on female employment in the first stage regression (S6 Table)—which is related to an increased walking time women need to reach the company. We argue for plausible exogeneity of the instrument as companies’ investment decisions are likely not related to women’s fertility decisions, but rather determined by immediate access to land, water and labor. Therefore the correlation between the instrument and unobserved differences in initial reproductive norms and female empowerment is likely very low (albeit not completely zero). We find that the coefficient on the predicted residuals in the second stage regression is insignificant (S5 Table), which is an indication that female employment is not endogenous. The insignificant effect of number of children in 2005 in the first stage regression (S6 Table) also points in this direction as it indicates that women’s fertility in 2005 did not influence the probability of employment and that reverse causality is not a major issue. In this case, the coefficient estimate for female employment in the 2SRI model is consistent but less efficient than the estimate of the standard Poisson regression. A Hausman test comparing the standard Poisson and 2SRI regressions does not reject the null hypothesis that the standard Poisson estimation is consistent and efficient.

The efficiency and consistency of this Poisson and IV approach importantly depends on the choice of the instrument. We argue for plausible exogeneity of the instrument but we acknowledge that the instrument, the distance to the nearest horticultural company, is not perfect. First, women with lower fertility preferences might move closer to the companies to access employment. This is not the case in our sample; only three women migrated after the companies established, which we consider negligible. Second, the presence of companies might influence fertility decisions through other channels than employment. Companies invest in infrastructure such as roads, school, and health centres as part of the land lease deals with the rural communities. This may affect fertility decisions irrespective of employment in the companies. Such effects are more indirect and likely less strong but could lead to an overestimation of the negative effect of employment on fertility (or an underestimation of the fertility-reducing effect of employment). However, these effects are at least partially captured by the variable ‘distance to concrete road’ in the Poisson regression model. Third, by choosing a location based on access to land, water and labor, companies might settle in areas with particular fertility rates. Companies may prefer to settle closer to the Senegal river, where villages could be more prosperous because of easy access to water; and this prosperity might have effected fertility decisions in the past. Companies may prefer to settle in villages with abundant labor resources; and this abundance might be related to high fertility rates in the past. Companies may settle in villages with stronger leadership because these villages are stronger in the negotiation process with the companies; and this may be related to different cultural fertility norms. Such unobserved effects may lead to some remaining bias in our estimates. However, these effects are at least partially captured by the variable ‘number of children in 2005’ in the Poisson regression model. Because of the difficulty to find a perfect instrument, we combine this method with the above described DD approach that does not depend on instruments but on recall information.

## Results

### Horticultural exports and female employment

Horticultural exports from Senegal increased from 5 million USD in 2003 to 45 million in 2011. The five export companies in the region Saint-Louis in the north of Senegal account for a major share of these exports. The increase in horticultural exports have created approximately 5,000 jobs in the region, of which 80% are occupied by women. The employees are mainly unskilled workers from the surrounding villages. The majority is hired as daily or seasonal worker; either as field worker for harvesting or as factory worker for washing, sorting and packing of produce. In the communities north of Saint Louis town (northern area), where the export companies are located because of easy access to irrigation water from the Senegal river, the share of women who are wage employed in the horticultural export sector increased from virtually zero in the early years 2000 to more than 20% in 2013 (Fig. 1a). In the neighboring communities south of Saint Louis town (southern area), where new investments in irrigation infrastructure and export companies are planned but not executed yet, there is a much smaller (only 3% in 2013) increase in the share of wage employed women (Fig. 1a). This can be explained by a larger distance to the companies, resulting in substantially more time needed for potential workers to reach the companies by foot. The road network density in the area is low and most people do not have any other means of transport. In addition, rural communities negotiated for preferential sourcing of labor from their own communities in the land lease deals with the companies.

From the 500 households that were surveyed, 132 have women that are employed for a wage outside the household and the family farm. The total income in these households is about 20% higher than in households without female employment (S1 Table). The wages of employed women contribute on average 23% to the total income of these households. These wages mainly (75%) come from the horticultural export industry but some women have other jobs (mainly domestic workers, hairdressers and garment-workers in Saint Louis town). In our sample, women are employed on average 6.7 months per year and 71% is employed for at least six months per year. Eighty-three percent of the employed women have a daily or seasonal contract while 17% have a yearly or permanent contract. During the employment period, women work nearly full-time with an average of 37 hours per week. Most employed women never worked outside the household and the family farm before their employment in export companies. Apart from wage employment, households obtain income from farming and small off-farm businesses. There are some differences between employed and non-employed women in observable individual, household and village characteristics (S2 Table). When analyzing the probability of women to be employed (S6 Table), we find the highest probability for ethnic non-Wolof, unmarried women around the age of 25 who live with their parents. Women in villages closer to the export companies and to the road network, and in villages with a single ethnicity and presence of female associations also have a higher probability of being employed. Women’s education and household asset ownership do not affect the likelihood of employment.

### Female employment and fertility

While female employment increased over time, fertility decreased. The number of births per woman was similar in the two areas in 2005 (0.15 for the northern area and 0.14 for the southern area) but decreased more sharply in the northern area (to 0.08 in 2013, compared to 0.11 for the southern area) where female employment increased most. The TFR, calculated as the sum of age-specific fertility rates for 5 year age cohorts for the period 2007–2012, is lower for the northern area (3.08) than for the southern area (3.61). It is difficult to compare the TFR calculations with the national TFR of 5.0 in Senegal, as they are based on a small number of observations and take only surviving children into account. Regional differences in TFR within the country likely exist but are not documented in secondary data. It is likely that the TFR in the Saint-Louis region is comparatively low, as it is a rather developed and less remote region. Yet, the difference in the calculated TFR between the two regions is an indication of true differences in fertility because they are calculated in the same way. The number of surviving children of employed women is substantially below that of unemployed women for age cohorts between 25 and 40 (Fig. 1b). Age at first marriage and age at first childbirth is higher for employed women than for unemployed women in the age cohorts 25–29 and 30–34 (Figs. 1c and 1d). For the age cohorts 18–24 and 35–40, there are no substantial differences in age at marriage and age at first childbirth between employed and unemployed women, likely because only a small share of the youngest women are married and have children and because the oldest women already had children before they started employment. By postponing marriage and first childbirth, employed women reduce the child-bearing years and the window of biological opportunity for subsequent children.

The results from the difference-in-differences (DD) estimation and the Poisson regression models can give us more insights into whether and to what extent the observed differences in fertility between employed and unemployed women can be attributed to the effect of female employment. The estimation results show that female employment significantly decreases the number of children, with point estimates varying between -0.22 to -0.33 (Table 1). The point estimates and estimated standard errors from the different models are quite similar, implying robust results. The IV approach results in similar point estimates but large standard errors, which is in line with consistent but less efficient estimates.

**Table 1:** Estimated effect of female employment on fertility from difference-in-differences and Poisson regression models. Source: own estimations from survey data.

	DD regression (coefficients)			Poisson regression (marginal effects)		
	DD	DD with covariates	DD with PSM	Poisson	Village FE	2SRI
Female employment	-0.332**	-0.291**	-0.320***	-0.256**	-0.215*	-0.289
	(0.139)	(0.122)	(0.130)	(0.109)	(0.125)	(0.557)

The reported results are summary results from full regression models that are presented in S3 and S5 Tables. The first column reports the simple DD regression. The second column reports the DD estimator when additional observable characteristics are taken into account. The third column reports the DD estimator after matching treated observations with untreated observations. The fourth column reports the average marginal effect of female employment on fertility from a cross-sectional Poisson regression, controlling for individual, household and village characteristics. The fifth column reports the average marginal effect of female employment on fertility from a cross-sectional Poisson regression, controlling for individual and household characteristics and village fixed effects. The last column reports the average marginal effect of female employment on fertility from a 2SRI model. Robust (column 1, 2, 4 and 5) and bootstrapped (column 3 and 6) standard errors are reported in parentheses. Significant effects are indicated with

* p<0.1.

** p<0.05.

*** p<0.01.

As the research area has a high prevalence of illiteracy (65% of women, S2 Table) and poverty (63% of households, S2 Table), we analyze how the effect of female employment on fertility changes with women’s education and with households’ poverty by including interaction terms in the cross-sectional Poisson model (Table 2). Illiterate women have significantly more children but the fertility-reducing effect of female employment is stronger for illiterate women. Employment reduces the number of children with 0.423 (or 23%) for illiterate women but has no effect for literate women. Women in poorer households have more children but the fertility reducing effect of female employment is as strong for women in poor households as for women in non-poor households.

**Table 2:** Estimated effect of female employment on fertility and changes in the effect with women’s literacy and household poverty from Poisson regression models. Source: own estimations from survey data.

Poisson (1)		Poisson (2)	
Female employment	-0.423***	Female employment	-0.297*
	(0.139)		(0.159)
Employment * literacy	0.462**	Employment * poverty	0.070
	(0.230)		(0.215)
Literacy	-0.342***	Poverty (MPI>33)	0.174**
	(0.114)		(0.087)
Other variables	Included	Other variables	Included

These results focus on the joint effect of female employment and literacy / poverty on fertility and are summary results from full regression models. The first column reports the average marginal effect of female employment and literacy on fertility from a cross-sectional Poisson regression, including an interaction term between employment and literacy, and controlling for individual, household and village characteristics. The second column reports the average marginal effect of female employment and poverty on fertility from a cross-sectional Poisson regression, including an interaction term between employment and poverty, and controlling for individual, household and village characteristics. Robust standard errors are reported in parentheses. Significant effects are indicated with

* p<0.1.

** p<0.05.

*** p<0.01.

### Other drivers of lower fertility

Apart from the employment status of women, other factors influence fertility as well, as can be revealed from the full regression results (S3 and S5 Tables). Older women, married women and Muslim women are found to have more children. Ethnicity has no significant effect on fertility. Women who are the wife of the household head and women in households with a female head or an older head of household have more children. Ownership of land and livestock, the main productive assets in the area, does not influence fertility. As already indicated above, women’s literacy promotes lower fertility while poverty increases fertility.

## Discussion and Conclusions

We find that female employment reduces the number of children per woman with 0.22 to 0.33. This is lower than the effects found for the Netherlands [18], China [20] and Kinshasa [21] but nevertheless still quite large. Relative to the sample average of 1.34 children per woman in the age category 18 to 40, this is a reduction of 16% to 25%. The effect is estimated only 8 years after rural women in the Saint Louis region faced a sudden opportunity for off-farm employment. For a substantial share of women considered in the analysis, fertility decisions were largely already taken when employment opportunities arose. If female employment persists or expands in the region, fertility is likely to reduce further if women are employed for a longer period and if younger women are employed before fertility decisions are taken.

We find that poverty and illiteracy increase the number of children per woman. This is in line with previous findings in the literature [21,30], and with the demographic-economic paradox of an inverse correlation between fertility and wealth [13]. We find a negative effect of female employment on fertility, when poverty and wealth are controlled for. This suggests that the fertility-reducing effect of female employment is not merely driven by an income effect. We find that the fertility-reducing effect of female employment is as strong for poor women as for non-poor women and stronger for illiterate women than for literate women. This points to the importance of a female empowerment effect. The results imply that employment is a strong instrument to empower poor and illiterate women.

Previous research has indicated that female employment in the horticultural export sector in Senegal increases the likelihood of primary-school-aged children, boys as well as girls, to be in school [31]. Combined with our results, this implies that female employment leads to investment in the quality of childcare rather than the quantity of children. This may lead to reinforcing effects in the long run. If female employment lowers fertility and increases girls’ education, and if women’s education is associated with reduced fertility, the effect may persist in the long run (even if exports and associated employment opportunities would stall) because daughters of employed women will be better educated and will have low-fertility preferences.

We provide evidence for a fertility-reducing effect of female employment in rural Senegal. Evidence for this link in rural areas of developing countries is non-existent but highly relevant given the high TFR in developing countries, SSA in particular, and the beneficial development effects associated with reduced fertility. Our results imply that employment in rural areas can be an important instrument for empowering women, reducing fertility and accelerating the demographic transition in poor countries. Our analysis contributes to the discussion on whether low-fertility preferences are the result of (collective) cultural or (individual) economic driving factors [6]. Our analysis is done at the micro-economic level, with individual women as unit of observation, and our results imply that fertility decreases (quite rapidly) through individual-specific economic changes. It is not unconceivable that female employment and its fertility-reducing effect at individual level, result in changes in reproductive norms in society and a (slower) cultural evolution towards low-fertility preferences. We did not address this issue as it requires a longer term perspective and a different analytical approach.

Our results are specific for the case-study region in Senegal. The fertility-reducing effect of female employment is likely impinged on by culture; which calls for caution in generalizing our results. Our research area has a high prevalence of polygamy and extended families living in compounds—which is to some extent characteristic for Western Africa but not for the rest of SSA. On the one hand, such a situation might ease female employment because there is less conflict between productive and reproductive tasks of women in the extended household—in our sample women from the same household are observed to take turns in working in export companies for a wage and staying home for reproductive tasks. The fertility-reducing effect of female employment might be rather modest in this case because labor substitution effects are less important. On the other hand, the empowerment of women in more traditional, extended and polygamist households is low [32]. A low initial bargaining power in the household might impede women to participate in off-farm employment but when they do, this employment might have a large effect on their autonomy. The fertility-reducing effect of female employment might be rather strong in this case because of a large empowerment effect.

The booming horticultural export sector represents the major source of off-farm wage employment for women in our study region. Our results imply that horticultural exports indirectly, through creating jobs accessible for women, contribute to a reduction in fertility rates. There is a large literature on the link between trade and development in general [33], and on the contribution of high-value food exports in particular [34], and a rising consensus that trade is good for development. Our findings corroborate this and add evidence for important indirect and non-monetary development effects of international trade and globalization.

Our findings have important policy implications. Many developing countries invest in family planning programs to slowdown population growth. While female employment contributes to reducing fertility by lowering fertility preferences, fertility may drop further if women who prefer fewer children are better aware of family planning methods and have better access to contraceptives. Family planning programs might therefore be more efficient and effective if targeted to areas with higher female labor participation rates or to employed women directly. Our results imply that employment in rural areas can have multiple and reinforcing effects on development. This calls for a recognition of the importance of labor markets, also in rural areas, in contemporary development thinking and policy.

## Supporting Information

S1 Dataset Dataset used for cross-sectional analysis. (DTA)

S2 Dataset Dataset used for difference-in-differences analysis. (DTA)

S1 Questionnaire Household survey conducted in April-June 2013 in Senegal River Delta. (PDF)

S1 Table Means comparison of income indicators for households with and without female employment. ***Source*: own calculations from survey data.** Comparisons are made between households with female wage employment and households without female wage employment using *t-*tests. Standard errors are reported in parentheses. Significant differences are indicated with * p<0.1, ** p<0.05 or *** p<0.01.
(PDF)

S2 Table Means comparison of individual, household and village characteristics for employed and non-employed women. ***Source*: own calculations from survey data.** Comparisons are made between wage employed women and non-wage employed women using t-tests. Significant differences are indicated with * p<0.1, ** p<0.05 or *** p<0.01. a One tropical livestock unit (TLU) equals 1 cow/horse, 0.8 donkey, and 0.2 sheep/goat. b The Multidimensional Poverty Index (MPI) is calculated according to the guidelines by the United Nations Development Program [28].
(PDF)

S3 Table Regression results of difference-in-differences estimations on fertility. ***Source*: own estimations from survey data.** The first column reports the simple DD regression *Y*_*i*_ *= β*_*0*_ *+β*_*1*_ *T*_*i*_ *+β*_*2*_ *t+β*_*3*_ *T*_*i*_ *t+ε*_*i*_ where *Y* is fertility, *T* is female employment and *t* is the year. The second column reports the DD regression when additional observable characteristics are taken into account: *Y*_*i*_ *= β*_*0*_ *+β*_*1*_ *T*_*i*_ *+β*_*2*_ *t+β*_*3*_ *T*_*i*_ *t+β*_*k*_ *X*_*ik*_ *+ε*_*i*_, where *X* is a vector of individual and household characteristics observed in 2005 and 2013. The third column reports the DD regression after matching treated observations with untreated observations based on an estimated propensity score and using Kernel matching, and controlling for *X*. The propensity score is estimated as the probability of employment conditional on *X*. Standard errors are reported in parentheses. Significant effects are indicated with * p<0.1, ** p<0.05 or *** p<0.01.
(PDF)

S4 Table Balancing properties of variables in treated and control groups for kernel matching on propensity scores. ***Source*: own estimations from survey data.** Balancing properties are tested so that pretreatment characteristics of treated and control units do not differ significantly after matching. Significant differences are indicated with * p<0.1, ** p<0.05 or *** p<0.01.
(PDF)

S5 Table Regression results of Poisson estimations on fertility. ***Source*: own estimations from survey data.** The first column reports the average marginal effects of the fertility determinants from a cross-sectional Poisson regression, controlling for individual, household and village characteristics. The second column reports the average marginal effects of the fertility determinants from a cross-sectional Poisson regression, controlling for individual and household characteristics and village fixed effects. The last column reports the average marginal effects of the fertility determinants from a 2SRI model. In the first stage, the distance to the nearest horticultural export company is used as an instrument for female employment. Standard errors are reported in parentheses. Significant effects are indicated with * p<0.1, ** p<0.05 or *** p<0.01.
(PDF)

S6 Table 2SRI estimation first stage regression results on the likelihood of employment. ***Source*: own estimations from survey data.** The table reports the Ordinary Least Square coefficient estimates of the first stage of the Two-stage Residual Inclusion. Probability of being employed is regressed on individual, household and village characteristics, and the distance to the nearest horticultural export company is used as instrument. Standard errors are reported in parentheses. Significant effects are indicated with * p<0.1, ** p<0.05 or *** p<0.01.
(PDF)
