# Supplementary material for: Female Employment Reduces Fertility in Rural Senegal
Source: PLoS One. 2015 Mar 27;10(3):e0122086. doi: 10.1371/journal.pone.0122086 (PMC4376695; doi:10.1371/journal.pone.0122086)
Supplement: S1 Questionnaire — (PDF) [file pone.0122086.s003.pdf]

ENQUETE AUPRES DES MENAGES – DELTA DU FLEUVE, SENEGAL

Avril – Juin 2013

Organisation :

Université Catholique de Louvain, la Belgique
Université Gaston Berger, le Sénégal

Responsables sur le terrain :

Goedele VAN DEN BROECK
Hannah PIETERS

Section A : Identification du ménage

A remplir avant et pendant l'interview

A1. Localisation : Village : ____ Communauté rurale :______________________________________

A2. Nom du chef du ménage : ____

A3. Numéro de téléphone : ____

A4. Est-ce que le ménage était interrogé dans l'enquête en 2006 ? **Vérifiez avec la liste des ménages.** 0 Oui [1] 0 Non [2] **Si oui :** Code d'identification du ménage: _____

Si non : Pourquoi pas :
0 Le village n'était pas interrogé en 2006 [1]
0 Le ménage ne peut pas être localisé / le ménage n'est pas présent dans le village [2]
0 Le ménage a été localisé mais ils refusent la coopération [3]

Si le ménage ne peut pas être localisé : pourquoi (demandez aux voisins) :
0 Le ménage a quitté le village temporairement / permanent [1]
0 Le ménage n'existe plus [2]
0 Autre raison (spécifiez) [3] : ___________________________________

A5. Nom du répondant des sections C à K : ____ Code ID

Nom de la répondante des sections L à N : ____ Code ID

Section B : Contrôle de l'opération

A remplir avant et après l'interview par les enquêteurs

B1. Date de l'interview _______ / _______/ 2013

B2. Nom de l'enquêteur ____

B3. Heure commencée _______ heures _______ minutes

Heure terminée _______ heures _______ minutes

Durée de l'interview _______ heures _______ minutes

A remplir après l'interview par les responsables

B4. Date de contrôle du questionnaire _______ / _______/ 2013

B5. Approuvé par le responsable (signature)

A remplir avant la saisie

B6. Nom de l'enregistreur ________________________________

Section C : Caractéristiques du ménage

Qui du ménage est le **chef du ménage (CM)** ? Qui est **responsable** de la **gestion quotidienne** du ménage et qui prend les **décisions journalières** sur les activités ?

Demandez à **parler** avec cette personne et notez le nom et le code ID du répondant à la page 1. De cette section jusqu'à section K les questions sont adressées à cette personne.

Expliquez la **définition** d'un **ménage** au répondant. Le ménage existe de tous les personnes qui **habituellement habitent, dorment et mangent** actuellement dans le ménage ou qui **habituellement habitaient, dormaient et mangeaient** dans le ménage pendant 6 mois ou plus pour la dernière année – qu'elles soient des parents ou pas.

C1. Combien est le nombre des **adultes** du ménage en total **(au moins 18 ans**) ? : ______ Rempliez le tableau ci-dessus et **commencez** avec le **CM**.

Membre du ménage	Nom	Sexe	Année de naissance	Lien avec le CM	Statut matrimo nial	**Si marié :** Dans quelle année s'est-il (elle) marié ?	Activité principale les derniers 12 mois	**Comprend** français (F) / arabe (A)?		Save-t-il (elle) **lire** français (F) / arabe (A)?		Save-t-il (elle) **écrire** français (F) / arabe (A) ?		Niveau de scolarité	Année de scolarité
Code ID		Code C1	année	Code C2	Code C3	année	Code C4	1=Oui 2=Non		1=Oui 2=Non		1=Oui 2=Non		Code C5	1/2/3/4/5/6
1				CM				F :	A :	F :	A :	F :	A :		
2								F :	A :	F :	A :	F :	A :		
3								F :	A :	F :	A :	F :	A :		
4								F :	A :	F :	A :	F :	A :		
5								F :	A :	F :	A :	F :	A :		
6								F :	A :	F :	A :	F :	A :		
7								F :	A :	F :	A :	F :	A :		
8								F :	A :	F :	A :	F :	A :		
9								F :	A :	F :	A :	F :	A :		
10								F :	A :	F :	A :	F :	A :		
11								F :	A :	F :	A :	F :	A :		
12								F :	A :	F :	A :	F :	A :		
13								F :	A :	F :	A :	F :	A :		
14								F :	A :	F :	A :	F :	A :		
15								F :	A :	F :	A :	F :	A :		
16								F :	A :	F :	A :	F :	A :		
17								F :	A :	F :	A :	F :	A :		
18								F :	A :	F :	A :	F :	A :		
19								F :	A :	F :	A :	F :	A :		
20								F :	A :	F :	A :	F :	A :		

CODE C1
1=homme
2=femme

CODE C2
1=conjoint
2=fils / fille
3=beau-fils / belle fille
4=petit-fils / petite-fille
5=neveu / nièce
6=cousin / cousine
7=parent / beaux parents
8=(beau)-frère / (belle)-sœur
9=autre (spécifiez)

CODE C3
1=marié
2=célibataire
3=veuf/veuve
4=divorcé
5=autre (spécifiez)

CODE C4
0=aucune
1=agriculture
2=élevage
3=pêche
4=commerce
5=artisanat
6=ouvrier agricole
7=employé
8=domestique
9=chauffeur/taxi moto
10=élève/étudiant
11=autre (spécifiez)

CODE C5
0=pas d'éducation
1=élémentaire
2=moyen
3=secondaire
4=technique
5=supérieure

C2. **Combien** est le **nombre** des **jeunes** du ménage en total (**entre 6 et 18 ans**) :

Membre du ménage	Nom	Sexe	Année de naissance	Lien avec le CM	Qui est le père ?	Qui est la mère ?	Statut matri-monial	**Si marié :** Dans quelle année ?	Activité principale les derniers 12 mois	Niveau de scolarité	Année de scolarité	L'intelligence de l'enfant	Inscrit cet année à l'école	**Si non :** La raison principale de ne pas être inscrit	**Si oui :** Dans le **mois passé**, combien de jours il (elle) était absent à l'école ?	**Si jours d'absence>0:** Raison principale pour être absent
Code ID		Code C1	année	Code C2	Code ID **(0 si père ne vit pas dans le ménage)**	Code ID **(0 si mère ne vit pas dans le ménage)**	Code C3	année	Code C4	Code C5	1/2/3/4/5/6	Code C6	1=Oui 2=Non	Code C7	Nombre de jours	Code C8
101																
102																
103																
104																
105																
106																
107																
108																
109																
110																
111																
112																
113																
114																
115																
116																
117																
118																
119																
120																

CODE C1
1=homme
2=femme

CODE C2
1=conjoint
2=fils / fille
3=beau-fils / belle fille
4=petit-fils / petite-fille
5=neveu / nièce
6=cousin / cousine
7=parent / beaux parents
8=(beau)-frère / (belle)-sœur
9=autre (spécifiez)

CODE C4
0=aucune
1=agriculture
2=élevage
3=pêche
4=commerce
5=artisanat
6=ouvrier agricole
7=employé
8=domestique
9=chauffeur/taxi moto
10=élève/étudiant
11=autre (spécifiez)

CODE C3
1=marié
2=célibataire
3=veuf/veuve
4=divorcé
5=autre (spécifiez)

CODE C5
0=pas d'éducation
1=élémentaire
2=moyen
3=secondaire
4=technique
5=supérieure

CODE C6
1= dessous de la moyenne
2=normal/moyenne
3= supérieur à la moyenne

CODE C7
1=incapable de payer scolarité
2=problèmes de transport/distance à l'école
3=mauvais résultats/difficultés à étudier
4=pas d'intéresse/motivation
5=études finalisées
6=travail domestique dans le ménage
7=travail aux champs du ménage
8=exclusion de l'école
9=problèmes de santé
10=autre (spécifiez)

CODE C8
1=problèmes de santé
2=incapable de payer scolarité
3=problèmes de transport/distance à l'école
4=raison familiale
5=garder les enfants/frères/sœurs
6=pas d'intéresse/motivation
7= travail aux champs du ménage
8=travail domestique dans le ménage
9= absence du maître/grève
10=autre (spécifiez)

C3. **Combien** est le **nombre** des **enfants** du ménage en total **(moins que 6 ans)** ?

Membre du ménage	Nom	Sexe	Année de naissance	Lien avec le CM	Qui est le père ?	Qui est la mère ?	L'enfant va à une crèche / pré-école ?
Code ID		Code C1	année	Code C2	Code ID **(0 si père ne vit pas dans le ménage)**	Code ID **(0 si mère ne vit pas dans le ménage)**	1=Oui 2=Non
201							
202							
203							
204							
205							
206							
207							
208							
209							
210							
211							
212							
213							
214							
215							
216							
217							
218							
219							
220							

CODE C1
1=homme
2=femme

CODE C2
1=conjoint
2=fils / fille
3=beau-fils / belle fille
4=petit-fils / petite-fille
5=neveu / nièce
6=cousin / cousine
7=parent / beaux parents
8=(beau)-frère / (belle)-sœur
9=autre (spécifiez)

C4. Quelle est **l'ethnie** du **CM**?
0 Oulof [1] 0 Peulh [2] 0 Serer [3] 0 Diola [4]
0 Maure [5] 0 Bambara [6] 0 Soninké / Sarakholé [7] 0 Manjack [8]
0 Lebou [9] 0 Autre ethnie sénégalaise [10] 0 Non sénégalais [11]

C5. Depuis **quand** le **CM habite**-t-il de façon **continue** dans cette **localisation ?** 0 Depuis la naissance [1] 0 Depuis : __________ (à préciser l'année) [2]

Si le CM n'est pas né[e] dans le village : **Où habitez**-vous **avant** de vous **installer ici ?** 0 Une ville [1] 0 Un village [2] 0 Etranger [3]

Pour quelle **raison principale** vous êtes-vous **installé** dans cette **localité** ?
0 Travail pour une entreprise horticole [1] 0 Autre travail [2] 0 l'Access à la terre [3] 0 Etude [4]
0 Raison familiale [5] 0 Autre raison (spécifiez) [6] : ______________________________

Est-ce que l'**installation** dans cette **localité** est 0 permanente [1] ou 0 temporaire [2]

C6. **Combien** est le **nombre** des **enfants du CM** qui **n'habitent plus** dans le ménage en total ?:

Membre du ménage	Nom	Sexe	Année de naissance	Qui est la mère ?	Statut matrimonial	**Si marié :** Dans quelle année s'est-il (elle) marié ?	Niveau de scolarité	Année de scolarité	Année dans laquelle il (elle) a quitté le ménage	Activité principale avant quitter le ménage	Raison principale pour quitter le ménage	Où habite-il (elle) actuellement ?
Code ID		Code C1	année	Code ID **(si mère ne vit pas dans le ménage : 0)**	Code C3	année	Code C5	1/2/3/4/5/6	année	Code C4	Code C9	Code C10
301												
302												
303												
304												
305												
306												
307												
308												
309												
310												
311												
312												
313												
314												
315												
316												
317												
318												
319												
320												

CODE C1
1=homme
2=femme

CODE C3
1=marié
2=célibataire
3=veuf/veuve
4=divorcé
5=autre (spécifiez)

CODE C4
0=aucune
1=agriculture
2=élevage
3=pêche
4=commerce
5=artisanat
6=ouvrier agricole
7=employé
8=domestique
9=chauffeur/taxi moto
10=élève/étudiant
11=autre (spécifiez)

CODE C5
0=pas d'éducation
1=élémentaire
2=moyen
3=secondaire
4=technique
5=supérieure

CODE C9
1=mariage
2=travail
3=études
4=accès à la terre
5=exclusion du ménage
6=disparu
7=autre (spécifiez)

CODE C10
1=même village où les parents habitent
2=un autre village
3=une ville
4=à l'étranger (en Afrique)
5=à l'étranger (dehors Afrique)
6=autre (spécifiez)

Section D : Emploi dans des entreprises horticoles

D1. Pendant **les 12 derniers mois**, est-ce que des **membres** du **ménage** ont été **impliqué** comme **salarier**, **saisonnier**, ou **journalier** dans une entreprise horticole (la GDS, SCL, SOCAS, STS, SOLDIVE ou autre)? 0 Oui [1] 0 Non [2] **Si non : Continuez à la suivante section E**

Si oui : Mentionnez tous les **membres** du **ménage** qui ont été impliqué pendant les **12 derniers mois** :

Membre du ménage	Chez quelle entreprise ?	Qu'est-ce qu'est l'activité principale ?	Avec quel statut ?	Depuis quand il / elle fait ça?	Il / elle a fait cet emploi :			Salaire reçu pour cet emploi ?		Revenu total de ces mois ?	Vous êtes engagé par un GIE ou par l'entreprise ?	Vous recevez le salaire d'un GIE ou de l'entreprise ?	**Si reçu d'un GIE :**	
					Combien de mois les 12 derniers mois ?	Combien de jours par mois en général ?	Combien d'heures par jour en général ?						Combien est-ce que vous devez payer au GIE ?	
Code ID	Code D1	Code D2	Code D3	année	mois	jours	heures	CFA	Code D4	CFA	Code D5	Code D5	CFA	Code D4

CODE D1
1=GDS
2=SCL
3=SOCAS
4=STS
5=SOLDIVE
6=Autre (spécifiez)

CODE D2
1=Production
2=Récolte
3=Conditionnement (transformation)
4=Gardien
5=Chauffeur
6=Technicien
7=Administration
8=Superviseur
9=Recruteur
10=Autre (spécifiez)

CODE D3
1=journalier
2=saisonnier
3=salarier
4=Autre (spécifiez)

CODE D4
1=par jour
2=par heure
3=par mois
4=par caisse/ cageot rempli
5=par ha
6=Autre (spécifiez)

CODE D5
1=GIE
2=entreprise

D2. Pouvez-vous indiquer la **certitude** du **travail** pour **chaque membre** du **ménagé** qui a été impliqué comme **salarier**, **saisonnier** ou **journalier** dans les **entreprises horticoles** pendant les **12 derniers mois** ? Les membres du ménage sont les **mêmes** comme au tableau de la question **D1**.
Si c'est possible, demandez à **chaque** membre du ménage qui travaille dans une entreprise horticole **personnellement** s'il est sûr de l'emploi.
Si un ouvrier n'est **pas présent**, demandez-le à la personne qui lui connaît le meilleur et **notez** le nom du **répondant** dans le tableau.

						**Si non :**		L'entreprise offre des services suivants ?			
Membre du ménage	Répondant	Toujours quand vous vouliez travailler dans une entreprise horticole, vous étiez employé et payé ?	**Si non :** Pourquoi pas ?	Le contrat est écrit ou oral ?	L'entreprise respecte toujours le contrat ?	Comment est-ce que l'entreprise n'a pas respecté le contrat principalement?	Combien de fois l'entreprise n'a pas respecté le contrat ?	Transport du village à l'entreprise	Repas	Soins de santé	Autre (spécifiez)
Code ID	Code ID	1=Oui 2=Non	Code D6	1=Ecrit 2=Oral	1=Oui 2=Non	Code D7	Nombre de fois	Code D8	Code D8	Code D8	Code D8

CODE D6
1=Il y avait déjà assez d'ouvriers
2=Je n'était pas assez qualifié (manque d'éducation)
3=Je n'était pas en bonne santé
4=J'étais discriminé pour mon ethnie
5=Autre (spécifiez)

CODE D7
1=Un salaire plus bas
2=Plus d'heures de travail
3=Pas de travail
4=Les conditions de sécurité/hygiène n'étaient pas respectées
5=Autre (spécifiez)

CODE D8
1=service offert gratuitement
2=service offert payant
3=service pas offert

D3. Pouvez-vous indiquer la **satisfaction** de **l'emploi** pour **chaque membre** du **ménagé** qui a été impliqué comme **salarier**, **saisonnier** ou **journalier** dans les **entreprises horticoles** pendant les **12 derniers mois** ? Les membres du ménage sont les **mêmes** comme au tableau de la question **D1**.
Si c'est possible, demandez à **chaque** membre du ménage qui travaille dans une entreprise horticole **personnellement** s'il est satisfait de l'emploi.
Si un ouvrier n'est **pas présent**, demandez-le à la personne qui lui connaît le meilleur et **notez** le nom du **répondant** dans le tableau.

Membre du ménage	Répondant	Etes-vous **satisfait** :						
		Du travail en **général** ?	Du statut ?	D'heures travaillés par jour ?	Du travail que vous devez faire ?	De l'environnement du travail (l'entreprise, autres ouvriers, ...)	Des services que l'entreprise offre ?	Du salaire ?
Code ID	Code ID	Code D9	Code D9	Code D9	Code D9	Code D9	Code D9	Code D9

CODE D9
1=Très satisfait
2=Satisfait
3=Plus ou moins satisfait
4=Ni satisfait, ni insatisfait
5=Plus ou moins insatisfait
6=Insatisfait
7=Très insatisfait

D4. Dans le **contrat** de l'emploi avec l'entreprise horticole / le GIE, est-ce qu'il y a des **attributs** que vous voudriez **changer** ? 0 Oui [1] 0 Non [2]

Si oui : Demandez à **chaque** membre du ménage qui travaille dans une entreprise horticole **personnellement** quels attributs est-ce qu'il voudrait changer.
Si un ouvrier n'est **pas présent**, demandez-le à la personne qui lui connaît le meilleur et **notez** le nom du **répondant** dans le tableau.

Membre du ménage	Répondant	Quels **attributs** est-ce que vous voudriez changer ?					
		Le salaire	**Si oui :** quel salaire par jour voudriez-vous ?	Le temps travaillé par jour	**Si oui :** combien de temps par jour voudriez-vous travailler ?	Le statut	**Si oui :** quel statut voudriez-vous ?
Code ID	Code ID	1=Oui 2=Non	CFA/jour	1=Oui 2=Non	Heures/jour	1=Oui 2=Non	Code D3

CODE D3
1=journalier
2=saisonnier
3=salarier
4=Autre (spécifiez)

D6. Grâce aux **salaires** que le ménage a gagné par **travailler pour des entreprises horticoles,** quelles **dépenses** suivantes sont devenues **possibles** ? **(Plusieurs réponses possibles)**

0 Une meilleure nourriture [1] 0 L'amélioration des maisons [2] 0 Les investissements dans l'agriculture [3] 0 Les investissements dans l'élevage [4]
0 Les investissements dans la pêche [5] 0 Une meilleure éducation pour les enfants [6] 0 Meilleures soins de santé [7]
0 Autre (spécifiez) [8] ____

Section E : Perception des entreprises horticoles

E1. Quelles entreprises horticoles est-ce que vous connaissez ? **(Plusieurs réponses possibles)** 0 GDS [1] 0 SCL [2] 0 SOCAS [3] 0 STS [4] 0 SOLDIVE [5]
0 Autre (spécifiez) [6] ____

E2. Est-ce que votre ménage sait s'il y a eu des réunions dans le village ou dans la CR où l'accès au foncier pour les entreprises horticoles était discuté ? 0 Oui [1] 0 Non [2]
Si oui : Rempliez le tableau :

Il y avait des réunions dans le village ?	Il y avait des réunions dans la CR ?	Qui de votre ménage a assisté à une réunion ? **(Plusieurs réponses possibles, code 0 si aucune personne a assisté)**	Pouvait-il (elle) intervenir dans les décisions ?	Un représentant des villageois a assisté à ces réunions?	**Si oui :** ce représentant pouvait intervenir dans les décisions ?
1=Oui 2=Non	1=Oui 2=Non	Code ID	1=Oui 2=Non	1=Oui 2=Non	1=Oui 2=Non

E3. Est-ce que vous percevez des **avantages** de la présence des **entreprises horticoles** dans la région? 0 Oui [1] 0 Non [2]
Si oui : Indiquez quels avantages :

E4. Est-ce que vous percevez des **désavantages** de la présence des **entreprises horticoles** ? 0 Oui [1] 0 Non [2]
Si oui : Indiquez quels désavantages

E5. Indiquez votre **degré d'accord** avec les **propositions** suivantes :

		Code E1
1	Les entreprises horticoles n'ont pas la droit d'exploiter des parcelles dans la CR pour l'exportation des produits.	
2	Les villageois sont impliqués dans le processus de l'accès au foncier aux entreprises horticoles.	
3	Le processus de l'accès au foncier aux entreprises horticoles se passe corrompu.	
4	Depuis que les entreprises se sont installées dans la région, il y a moins de terres disponibles pour les paysans où ils peuvent cultiver.	
5	Depuis que les entreprises se sont installées dans la région, il y a moins de terres disponibles pour les éleveurs où leur bétail peut brouter.	
6	Les entreprises horticoles prennent des terres des paysans.	
7	Les entreprises horticoles prennent des terres des éleveurs.	
8	Les entreprises horticoles utilisent beaucoup d'eau afin qu'il y ait moins de l'eau pour les paysans.	
9	Les entreprises horticoles utilisent beaucoup de pesticides ce qu'est négative pour l'environnement.	
10	Les entreprises horticoles utilisent beaucoup de pesticides ce qu'est négative pour la santé des villageois.	
11	Les entreprises horticoles devraient payer plus de taxes pour l'exploitation des terres.	
12	Les entreprises horticoles devraient payer plus de taxes pour l'utilisation de l'eau.	
13	Les entreprises horticoles créent beaucoup d'emploi ce qu'est bon pour les villageois.	
14	Les entreprises horticoles investissent beaucoup dans des initiatives locales pour le développement rural.	
15	Les entreprises horticoles exploitent des ouvriers et utilisent la population locale comme main d'œuvre pas chère.	
16	Depuis que les entreprises horticoles se sont installées dans la région, on observe une amélioration dans les conditions de vie des villageois.	
17	Les entreprises horticoles produisent pour l'exportation afin qu'il y ait moins de nourriture disponible dans la région pour la population locale.	
18	Depuis que les entreprises horticoles se sont installées dans la région, la façon de vivre traditionnel a changée.	
19	Plus de directeurs des entreprises horticoles devraient avoir la nationalité Sénégalaise, et pas être originaire de l'étranger.	
20	Le siège social des entreprises horticoles doivent être dans les CR.	
21	Avant que les entreprises horticoles se sont installées dans la région, il n'y avait pas assez d'emploi.	
22	En général, je suis content avec la présence des entreprises horticoles dans la région.	
23	Dans le futur, je voudrais avoir plus d'entreprises horticoles dans la région.	

CODE E1
1=Tout à fait d'accord
2=D'accord
3=Plus ou moins d'accord
4=Neutre
5=Plus ou moins pas d'accord
6=Pas d'accord
7=Pas du tout d'accord
8=Ne sait pas

Section F : Terres Agricoles

F1. Est-ce que le **ménage possède** / **exploite** des **terres** agricoles **actuellement**? 0 Oui [1] 0 Non [2]

Si oui : Combien de **parcelles** sont **possédées** par le ménage **actuellement**? ☐ Combien de **parcelles** sont **exploitées** par le ménage (**les 12 derniers mois)**? ☐

Combien est la **superficie totale possédée** par le ménage **actuellement**? ☐ **ha** et la **superficie totale exploitée** par le ménage (**les 12 derniers mois)**? ☐ **ha**

F2. **Il y a 5 ans**, est-ce que le **ménage possédait** / **exploitait** des **terres** agricoles ? 0 Oui [1] 0 Non [2]

Si oui : Combien de **parcelles** étaient **possédées** par le ménage **il y a 5 ans**? ☐ Combien de **parcelles** étaient **exploitées** par le ménage **(il y a 5 ans)**? ☐

Combien était la **superficie totale possédée** par le ménage **il y a 5 ans**? ☐ **ha** et la **superficie totale exploitée** par le ménage **(il y a 5 ans)**? ☐ **ha**

F3. **Il y a 10 ans**, est-ce que le **ménage possédait** / **exploitait** des **terres** agricoles ? 0 Oui [1] 0 Non [2]

Si oui : Combien de **parcelles** étaient **possédées** par le ménage **il y a 10 ans**? ☐ Combien de **parcelles** étaient **exploitées** par le ménage **(il y a 10 ans)**? ☐

Combien était la **superficie totale possédée** par le ménage **il y a 10 ans**? ☐ **ha** et la **superficie totale exploitée** par le ménage **(il y a 10 ans)**? ☐ **ha**

F4. Pouvez-vous **énumérer** toutes les **parcelles possédées** ou/et **exploitées** par le ménage pendant **les 12 derniers mois**:

No parcelle	Superficie de la parcelle	A quelle distance du logement se trouve cette parcelle ?	Combien de temps pour se transporter à la parcelle ?	Quel type d'irrigation est utilisé sur cette parcelle ?	Quand avez-vous commencé à utiliser ce système d'irrigation ?	Quel type d'eau / de forage est utilisé pour cette parcelle ?	Comment percevez-vous la qualité du sol ?
	ha	km	min	Code F1	année	Code F2	Code F3
1							
2							
3							
4							
5							
6							
7							
8							
9							
10							

CODE F1
0=exploitation pluviale
1=irrigation manuelle (par arrosoirs / seaux)
2=irrigation par lance
3=irrigation par GMP (groupement motopompes)
4=irrigation par aspersion
5=irrigation goutte-à-goutte
6=irrigation gravitaire
7=Autre (spécifiez)

CODE F2
1=eau SDE
2=puits traditionnelles (séans)
3=puits améliorés / busés
4=forage collectif, pompage manuelle
5=forage collectif, pompage électrique
6=forage individuel, pompage manuelle
7=forage individuel, pompage électrique
8=Autre (spécifiez)

CODE F3
1=très riche
2=riche
3=moyen
4=pauvre
5=très pauvre

F5. Pouvez-vous décrire pour toutes les **parcelles possédées** ou/et **exploitées** par le ménage pendant **les 12 derniers mois l'accès au foncier :**
Ce sont les mêmes parcelles comme dans le tableau F4 !

No parcelle	Le ménage est propriétaire de la parcelle ?	**Si le ménage est propriétaire :** Qui est le propriétaire ?	Le ménage exploite la parcelle ?	**Si le ménage n'est pas propriétaire :** Le ménage loue en espèces/nature/gratuit ?	Qui exploite cette parcelle principalement ?	Comment le ménage a obtenu la parcelle ?	**Si c'est par l'achat :** Prix payé pour acheter la parcelle ?	**Si c'est par location/ bail :** Pendant **les 12 derniers mois**, prix payé aux bailleurs?	Depuis quelle année la parcelle a été attribuée au ménage ?	La parcelle est enregistrée ?	**Si oui :** depuis quand ?
	1=Oui 2=Non	Code ID	Code F4	Code F5	Code ID	Code F6	CFA	CFA	année	1=Oui 2=Non	année
1											
2											
3											
4											
5											
6											
7											
8											
9											
10											

CODE F4
1=Exploite lui-même
2=Baillée en espèces
3=Baillée en nature (métayage)
4=Parcelle pas cultivée
5=Autre (spécifiez)

CODE F5
1=Loue en espèces
2=Loue en nature (métayage)
3=Utilise la parcelle à titre gratuite/terre communale
4=Autre (spécifiez)

CODE F6
1=l'achat
2=location en espèce
3=location en nature
4=transfert à titre gratuit
5=héritage/dot
6=Autre (spécifiez)

F6. Est-ce que **vous** voulez **augmenter** la **superficie** total de terres agricoles ? 0 Oui [1] 0 Non [2]
Si oui : Est-ce que c'est **possible** que vous augmentez la superficie total de terres agricoles ? 0 Oui [1] 0 Non [2]
Si non : Pourquoi est-ce que ce n'est pas possible ? 0 Pas assez d'argent [1] 0 Pas assez de terres disponibles [2] 0 Autre (spécifiez) [4] _________________

F7. Est-ce que **vos enfants** veulent **augmenter** la **superficie** total de terres agricoles pour leur même production agricole ? 0 Oui [1] 0 Non [2]
Si oui : Est-ce que c'est **possible** que vos enfants augmentent la superficie total de terres agricoles ? 0 Oui [1] 0 Non [2]
Si non : Pourquoi est-ce que ce n'est pas possible ? 0 Pas assez d'argent [1] 0 Pas assez de terres disponibles [2] 0 Autre (spécifiez) [4] __________________

F8. **Pendant les 10 dernières années**, est-ce que le ménage a **transféré**, **rendu** ou **perdu** les **droits d'exploitation** des terres ? 0 Oui [1] 0 Non [2]
Si oui : Combien de **parcelles** en total ? ☐ Et combien est la **superficie totale** ? ☐ **ha**
Rempliez le tableau ci-dessus :

No parcelle	Superficie de la parcelle	Dans quelle année c'est transférée/rendue/perdue ?	Qui exploite ça actuellement?	Reçu une compensation ?	**Si oui :** Combien était la compensation en total?
	ha	année	Code F3	1=Oui 2=Non	CFA
11					
12					
13					
14					
15					

CODE F3
0=Pas d'exploitation
1=Famille
2=Autres paysans
3=CR
4=Entreprise horticole
5=Autre (spécifiez)

Section G: Production agricole

G1. Est-ce que le **ménage** a **cultivé** et/ou **récolté** des **produits** comme **exploitant** pendant **les 12 derniers mois** ? 0 Oui [1] 0 Non [2] **Si non : continuez à la section H**

G2. Est-ce que le **ménage** a **cultivé** et/ou **récolté** des **produits** comme **exploitant** pendant **la contre-saison froide** 2012 / 2013 (d'octobre 2012 à février 2013) ?

0 Oui [1] 0 Non [2] **Si oui : Rempliez le tableau ci-dessus**

Culture	Qui était responsable pour cette culture?	Superficie cultivée	Quantité de production total		La quantité destinée pour le ménage		La quantité destinée pour la vente		Le prix unitaire de la vente ?		Valeur totale des ventes	Mois de récolte
Code G1	Code ID	ha		Code G2		Code G2		Code G2	CFA	Code G2	CFA	Code G3

G3. Est-ce que le **ménage** a **cultivé** et/ou **récolté** des **produits** comme **exploitant** pendant **l'hivernage** 2012 (de juin 2012 à septembre 2012) ?

0 Oui [1] 0 Non [2] **Si oui : Rempliez le tableau ci-dessus**

Culture	Qui était responsable pour cette culture?	Superficie cultivée	Quantité de production total		La quantité destinée pour le ménage		La quantité destinée pour la vente		Le prix unitaire de la vente ?		Valeur totale des ventes	Mois de récolte
Code G1	Code ID	ha		Code G2		Code G2		Code G2	CFA	Code G2	CFA	Code G3

G4. Est-ce que le **ménage** a **cultivé** et/ou **récolté** des **produits** comme **exploitant** pendant **la contre-saison chaude** 2013 (de mars 2013 à juin 2013) ?

0 Oui [1] 0 Non [2] **Si oui : Rempliez le tableau ci-dessus**

(Si la contre-saison chaude 2013 n'est pas encore finit, demandez pour la contre-saison chaude **2012** (de mars 2012 à juin 2012) – et indiquez le cas)

0 contre-saison chaude 2013 0 contre-saison chaude 2012

Culture	Qui était responsable pour cette culture?	Superficie cultivée	Quantité de production total		La quantité destinée pour le ménage		La quantité destinée pour la vente		Le prix unitaire de la vente ?		Valeur totale des ventes	Mois de récolte
Code G1	Code ID	ha		Code G2		Code G2		Code G2	CFA	Code G2	CFA	Code G3

CODE G1

1=Riz
2=Tomate industrielle
3=Tomate cerise
4=Autre tomates
5=Haricots verts
6=Haricots niebes
7=Oignon
8=Chou
9=Carottes
10=Poivrons
11=Aubergines
12=Pomme de terre
13=Patate douce
14=Gombo
15=Mangue
16=Bananes
17=Melon
18=Pastèque
19=Mais
20=Arachide
21=Mil
22=Autre (spécifiez)

CODE G2

1=par kg
2=par cageot
3=par carton
4=par filets
5=par sac
6=par botte
7=autre (spécifiez

CODE G3

1=janvier
2=février
3=mars
4=avril
5=mai
6=juin
7=juillet
8=aout
9=septembre
10=octobre
11=novembre
12=décembre

G5. Pouvez-vous **mentionner** toutes les **dépenses** pour la **production**, la **transformation** et la **commercialisation** des **produits** agricoles **pendant les 12 derniers mois** ci-dessus :

N° de dépense	Type de dépense	Contre-saison froide		Hivernage		Contre-saison chaude	
		Quantité applique en total	Dépenses totales	Quantité applique en total	Dépenses totales	Quantité applique en total	Dépenses totales
			CFA		CFA		CFA
1	Semences	kg		kg		kg	
2	Produits phytosanitaires	l		l		l	
3	Engrais	kg		kg		kg	
4	Eau d'irrigation						
5	Location et entretien des machines et équipements						
6	Services agricoles / prestations						
7	Transport / carburant / électricité / matériel						
8	Autre dépenses (spécifiez) :						

G6. Pouvez-vous **mentionner** tous les **membres** du ménage qui ont **travaillé** dans la **production**, **transformation** et **commercialisation** des **produits** agricoles **pendant les 12 derniers mois** ci-dessus :

Membre du ménage	Combien de mois il/elle a travaillé pour ces activités?	En général pour ces activités :	
		Combien de jours par mois il/elle travaille ?	Combien d'heures par jour il/elle travaille ?
Code ID	mois	jours	heures

G7. Est-ce que le ménage a utilisé pour la production mentionné ci-dessus de **main d'œuvre non-familiale** (des journaliers, saisonniers, récolteuses, etc.) **pendant les 12 derniers mois**?
0 Oui [1] 0 Non [2]

Si oui : **Personnes-jours** de **main d'œuvre** utilisée : ______ personnes-jours

Combien avez-vous payé cette main d'œuvre : ______ CFA par 0 heure [1] 0 jour [2] 0 mois [3] 0 autre (spécifiez) [4]:

Dépenses totales des **salaires** de cette **main d'œuvre** : ______ CFA

Autres dépenses pour cette main d'œuvre (**repas**, **transport**, etc) : ______ CFA

G8. **Connaissez**-vous le **Global GAP** ? 0 Oui [1] 0 Non [2]

G9. **Indiquez** si vous appliquez les **pratiques** suivantes aux propres terres agricoles :

N° de pratique		Appliquez-vous cette pratique dans votre ferme ?	Si oui : Depuis quand ?	Pour quelles cultures ? **(Plusieurs réponses possibles)**
		1=Oui 2=Non	Année **(0 si plus de 10 ans)**	Code G1
1	Semences améliorés			
2	Engrais chimique			
3	Engrais organique			
4	Tenir un registre de l'engrais appliqué			
5	Compostage des matériaux organiques et utilisation du compost aux champs			
6	Produits phytosanitaires			
7	Tenir un registre des produits phytosanitaires appliqués			
8	Rotation des cultures			
9	Semis en ligne			
10	Comptabilité de la production agricole des revenus et des dépenses			
11	Laver les mains après l'utilisation d'engrais et des produits phytosanitaires			
12	Ne pas appliquer les produits phytosanitaires juste avant la récolte			
13	Stockage séparé des semences et des produits phytosanitaires			
14	Suivre une itinéraire technique			
15	Porter des vêtements protecteurs pendant appliquer des produits chimiques			
16	Ne pas laisser des déchets aux champs			
17	Clôture des champs contre des animaux			

CODE G1
1=Riz
2=Tomate industrielle
3=Tomate cerise
4=Autre tomates
5=Haricots verts
6=Haricots niebes
7=Oignon
8=Chou
9=Carottes
10=Poivrons
11=Aubergines
12=Pomme de terre
13=Patate douce
14=Gombo
15=Mangue
16=Bananes
17=Melon
18=Pastèque
19=Mais
20=Arachide
21=Mil
22=Autre (spécifiez)

G10. Le ménage cultive-t-il **la tomate industrielle sous contrat** avec la SOCAS **actuellement**? 0 Oui [1] 0 Non [2] **Si Oui : remplicz le tableau ci-dessus**

Depuis quand ?	Pour la dernière campagne :		
	Superficie sous contrat ?	Quantité fournie à SOCAS ?	Prix reçu ?
année	ha	kg	CFA par kg

G11. Le **ménage** a-t-il **jamais eu** un **contrat** (**pour la tomate industrielle avec SOCAS) ?** 0 Oui [1] 0 Non [2] **Si Oui : remplicz le tableau ci-dessus**

En quelle année commencé ?	En quelle année arrêté ?	Superficie sous contrat ?	Qui a terminé le contrat ?
année	année	ha	1=ménage 2=SOCAS 3=GIE

G12. **Si le ménage n'a jamais eu un contrat** : **Voudriez**-vous **cultiver** la tomate industrielle sous **contrat ?** 0 Oui [1] 0 Non [2]
Avez-vous **déjà essayé** d'avoir accès a un **contrat ?** 0 Oui [1] 0 Non [2]

G13. Pendant les **dernières années**, est-ce que vous avez eu un **problème** de **manque d'eau** pour les cultures ? 0 Oui [1] 0 Non [2]

Si oui : Quels étaient les **conséquences** : ____________________

G14. Pendant les **dernières années**, est-ce que vous avez eu un **problème** avec la **qualité d'eau** pour les cultures ? 0 Oui [1] 0 Non [2]

Si oui : Quels étaient les **problèmes** :____________________

G15. Pendant les **dernières années**, est-ce que vous pensez que la **pluie** a **changé** ? 0 Oui [1] 0 Non [2]

Si oui : Comment est-ce que la pluie a changé ? 0 Moins de pluie [1] 0 Plus de pluie [2] 0 Parfois moins de pluie, parfois plus de pluie [3]
0 Autre (spécifiez) [4] ____________________

G16. Pendant les **dernières années**, est-ce que vous pensez que la **richesse du sol** a **changé** ? 0 Oui [1] 0 Non [2]

Si oui : Comment est-ce que la richesse du sol a changé ? 0 Amélioré [1] 0 Détérioré [2] 0 Autre (spécifiez) [3] ____________________

Section H : l'Elevage

H1. Le **ménage possède** du **bétail actuellement**? 0 Oui [1] 0 Non [2] **Si non : continuez à la suivante section I**

Mentionnez tous les **animaux possédés actuellement** et indiquez si le ménage a vendu et / ou **abattu des animaux du propre bétail**, pendant les **12 derniers mois**.

Animal	Nombre d'animaux possédés **actuellement**	A qui appartiennent ces animaux ?	Nombre d'animaux vendus pendant les **12 derniers mois**	**Si nombre d'animaux vendus>0 :** Prix moyen par animal vendu	Nombre d'animaux abattus pour propre consommation pendant **les 12 derniers mois**
Code H1		Code ID		CFA	

CODE H1
1=Vache / bovins
2=Cheval / équins
3=Ane / azins
4=Chèvre / caprins
5=Mouton / ovins
6=Poulet / volaille
7=Canard
8=Dromadaire / Chameau
9=Autre (spécifiez) :

H2. Pendant **le dernier mois**, votre **bétail** a produit des **produits animaux** (comme du lait, des œufs, des peaux/cuir, du fumier) **?**: 0 Oui [1] 0 Non [2]

Si oui : Remplissez le tableau ci-dessus :

N° de produit	Produits animaux	Quantité consommée /utilisée dans le ménage		Quantité vendue		Prix unitaire reçu pour ces ventes ?	
						CFA	par
1	Du lait		l		l		l
2	Des œufs		Œufs		Œufs		Œufs
3	Des peaux / cuir		Peau		Peau		Peau
4	Du fumier / fiente volaille		Kg		Kg		Kg
5	Autre produits (spécifiez) :						

H3. Pendant les **12 derniers mois**, le **bétail** a **pâturé** aux champs ? 0 Oui [1] 0 Non [2]

Si oui : Remplissez le tableau ci-dessus :

Le propriétaire des champs	Pâturent-ils sur les champs ?	**Si entreprises horticoles :** Quelle entreprise horticole ?	**Si oui :** En quels mois pâturent-ils? **(Indiquez avec un x)**												Combien devez-vous payer en total?	Comment percevez-vous la qualité de la nourriture ?	Quels animaux pâturent-ils ?
	1=Oui 2=Non	Code H2	Jan	Fév	Mar	Avril	Mai	Juin	Jui	Aout	Sep	Oct	Nov	Déc	CFA	Code H3	Code H1
1. Propre terre																	
2.Autres paysans																	
3.Village																	
4.Communauté Rurale																	
5.Terrains communes																	
6.Entreprises horticoles																	
7.Autre (spécifiez) :																	

CODE H2
1=GDS
2=SCL
3=SOCAS
4=STS
5=SOLDIVE
6=Autre (spécifiez)

CODE H3
1=très riche
2=riche
3=moyen
4=pauvre
5=très pauvre

H4. Est-ce que vous avez **acheté** de la **nourriture** pour tout le **bétail** pendant les **12 derniers mois** : 0 Oui [1] 0 Non [2]

Si oui : Remplissez le tableau ci-dessus :

Quel type de nourriture?	Où achetez-vous ?	Combien achetez-vous en total?	Prix unitaire pour l'achat ?	Dépenses totales pour la nourriture ?	Pour quels animaux ? **(Plusieurs réponses possibles)**	Comment percevez-vous la qualité de la nourriture ?
Code H4	Code H5	kg	CFA/kg	CFA	Code H1	Code H3

CODE H4
1=paille d'arachide
2=paille de mil
3=paille de riz
4=son de mil
5=son de riz
6 =aliment industriel
7=l'herbe sauvage
8=autre (spécifiez)

CODE H1
1=Vache / bovins
2=Cheval / équins
3=Ane / azins
4=Chèvre / caprins
5=Mouton / ovins
6=Poulet / volaille
7=Canard
8=Dromadaire / Chameau
9=Autre (spécifiez) :

CODE H3
1=très riche
2=riche
3=moyen
4=pauvre
5=très pauvre

CODE H5
1=commerçants
2= entreprises horticoles
3=autres paysans
4=Autre (spécifiez)

H5. Pendant **le dernier mois**, **combien** étaient les **dépenses** pour le **bétail** et la **commercialisation** des produits animaux :

- Services vétérinaires / vaccins / médicaments ______ CFA
- Le transport / carburant ______ CFA
- Entretien / location / réparation des écuries ______ CFA
- Salaires / dépenses pour des bergers embauchés ______ CFA
- Autre dépenses (spécifiez) : ________________________ ______ CFA

H6. Mentionnez **tous** les **membres** du **ménage** qui ont **travaillé** pour **garder** le **bétail**, la **transformation** et la commercialisation des **produits animaux** pendant **les 12 derniers mois** :

Membre du ménage	Combien de mois il/elle a travaillé pour ces activités?	En général pour ces activités :	
		Combien de jours par mois il/elle travaille	Combien d'heures par jour il/elle travaille
Code ID	mois	jours	heures

Section I: Le crédit

I1. Pendant les **12 derniers mois**, est-ce que le **ménage** a-t-il **pris** du **crédit**? 0 Oui [1] 0 Non [2]

Si oui: Pourriez vous **mentionner** tous les **emprunts** pendant les **12 derniers mois :**

Source de crédit	Le montant emprunté	Qui a pris le crédit ?	Taux d'intérêt ?	
Code I1	CFA	Code ID	%	Code I2

CODE I1
1=CNCA – Caisse Nationale de Crédit Agricole
2=ACEP – Alliance du Crédit et de l'épargne pour la production
3=CPEC – Caisses populaires d'épargne et de crédit
4=MEC – Mutuelle d'épargne et de crédit
5=Autre institution (spécifiez)
6=des prêteurs (sur gages)
7=des commerçants
8=la famille / des amis / des voisins
9=tontine
10=Autre source (spécifiez)

CODE I2
1=par semaine
2=par mois
3=par année

I2. **Vouliez**-vous (**ou quelqu'un** du **ménage**) **prendre du** (**plus** de) **crédit** pendant des **12 derniers mois?** 0 Oui [1] 0 Non [2]

I3. **Avez**-vous (**ou quelqu'un** du **ménage**) **sollicité du** (**plus** de) **crédit** pendant des **12 derniers mois mais** essuyé un **refus?** 0 Oui [1] 0 Non [2]

I4. **Qu'est ce** que vous **pensez** de l'**accès** au **crédit** pour **votre ménage?** 0 très difficile [1] 0 difficile [2] 0 ni difficile, ni facile [3] 0 facile [4] 0 très facile [5]

Si difficile/très difficile : Quelle est la **raison principale?** 0 trop loin [1] 0 trop chère [2] 0 manque de garanti [3] 0 autre (spécifiez) [4] __________________

Section J : Autres sources de revenu

J1. Pendant **les 12 derniers mois**, est-ce que des **membres** du **ménage** ont été impliqués dans des **activités non-agricoles** comme salarier, saisonnier ou journalier (e.g. **ouvrier** dans une **usine**, **bonne**, **fonctionnaire**, etc.?

0 Oui [1] 0 Non [2] **Si oui** : **Mentionnez** tous les **membres** du **ménage** qui ont été impliqué pendant les **12 derniers mois :**

Membre du ménage	Quel type de travail ?	Avec quel statut ?	A quelle distance se trouve l'emploi?	Il / elle a fait cet emploi : Combien de mois les 12 derniers mois ?	Combien de jours par mois en général ?	Combien d'heures par jour en général ?	Salaire reçu pour cet emploi ?		Revenu total de ces mois ?
Code ID	Code J1	Code J2	km	mois	jours	heures	CFA	Code J3	CFA

CODE J1
1=usine
2=bonne
3=fonctionnaire
4=Autre (spécifiez)

CODE J2
1=journalier
2=saisonnier
3=salarier
4=Autre (spécifiez)

J2. Pendant **les 12 derniers mois**, est-ce que des **membres** du **ménage** ont fait **des affaires / activités non-agricoles autonomes** qui produisent des revenus pour le ménage ?
E.g. commerce, **artisanat**, **chauffeur**, **coiffeuse**, tailleur, maçon, mécanicien, etc. 0 Oui [1] 0 Non [2]

Si oui : **Mentionnez** tous les **membres** du **ménage** qui ont été impliqué pendant les **12 derniers mois** :

Membre du ménage	Type d'affaires	Depuis quand ?	Il / elle a fait ces affaires : Combien de mois les 12 derniers mois ?	Combien de jours par mois en général ?	Combien d'heures par jour en général ?	Pour **les 12 derniers mois** : Revenu total de ces affaires ?	Les dépenses totales de ces affaires ?
Code ID	Code J4	année	mois	jours	heures	CFA	CFA

CODE J3
1=par jour
2=par heure
3=par mois
4=Autre (spécifiez)

CODE J4
1=Commerçant
2=Chauffeur
3=Maçon
4=Charpentier
5=Bûcheron
6=Mécanicien
7=Coiffeuse
8=Tailleur
9=Pêcheur
9=Autre (spécifiez)

J3. Pendant **les derniers 12 mois**, combien de revenu le ménage a reçu de :

		Revenus des **12 derniers mois** ?
		CFA
1	Des remises et transferts d'une personne	
2	Dot, héritage	
3	Location des machines, équipements, véhicules, animaux	
4	Location des maisons / autres bâtiments	
5	Intérêt d'épargne ou crédit offert ?	
6	Pension, subventions, transferts du gouvernement / organisation	
7	Cadeaux, loterie, prix, etc.	
8	Contributions sociales (zakat)	
9	Autre source de revenu pas encore mentionné (spécifiez) :	

Section K : Capitaux et biens du ménage

K1. **Combien** de m^2 est la **superficie** (des bâtiments) du **logement actuel**? [ ] **m^2**

Nombre de **pièces** pour **dormir** dans le logement ? [ ] **pièces**

K2. Quel est le **principal matériel** du **sol** du logement ? 0 Terre /sable [1] 0 Bouse [2] 0 Parquet / Bois de cure [3] 0 Bande de vinyle / tapis [4] 0 Carrelage [5] 0 Moquette [6] 0 Ciment [7] 0 Autre (spécifiez) [8] ______________________

Quel est le **principal matériel** des **murs** du logement ? 0 Béton / briques / pierre [1] 0 Fer galvanisé [2] 0 Bois [3] 0 Feuilles / branches / paille [4] 0 La bouse [5] 0 Autre (spécifiez) [6] ______________________

K3. Quel est le **principal mode** d'**éclairage pour** votre **ménage actuellement ?** 0 Bois [1] 0 Pétrole [2] 0 Gaz [3] 0 Bougie [4] 0 Electricité / énergie solaire [5] 0 Autre (spécifiez) [6] ______________________

K4. Quel est le **principal combustible** pour la **cuisine** maintenant ? 0 Bois / charbon de bois [1] 0 Pétrole [2] 0 Gaz [3] 0 Electricité / énergie solaire [4] 0 Bouse de vache/d'animaux [5] 0 Autre (spécifiez) [6] ______________________

K5. Quelle est la **source principale** d'**eau à boire** pour les **membres** de votre **ménage ?**

0 Eau du robinet dans le logement [1**]** 0 Eau du robinet dehors du logement [2] 0 Borne fontaine [3] 0 Puits à pompe [4]
0 Puits protégés [5] 0 Fut [tonneau] [6] 0 Eau de pluie [7] 0 Eau en bouteille [8]
0 Puits non protégés [9] 0 Mare, ruisseau ou fleuve [10] 0 Canal [11] 0 Camion citerne, vendeur d'eau [12]
0 Forage [13] 0 Autre (spécifiez) [14] ______________________

K6. Quelle **distance** faut-il **parcourir** pour **aller** jusqu'au **lieu** où se **trouve** cette **eau ?** 0 Sur place [1] 0 A moins de 100 mètres [2] 0 Entre 100 et 500 mètres [3] 0 Entre 500 mètres et 1km [4] 0 A plus de 1 km [5]

K7. Quel **type** de **toilette** utilisez-vous ? 0 Toilette à chasse [1] 0 Toilette à trou [2] 0 Latrine puisard [3] 0 Pas de toilette [4] 0 Autre (spécifiez) [5] ______

K8. **Combien** de **fois par jour** lavez-vous vos **mains** avec **savon** ou un **autre produit antibactérien** ? 0 Jamais [1] 0 Une fois par jour [2] 0 2 à 3 fois par jour [3] 0 Plus que 3 fois par jour [4]

K9. Il y a un **branchement** au **secteur** de **l'électricité** dans le **logement** ? 0 Oui [1] 0 Non [2]

K10. Est-ce que le **ménage actuellement possède** des **objets** suivants ?

N° d'actif	Type d'actif	Nombre actuellement possédé	N° d'actif	Type d'actif	Nombre actuellement possédé	N° d'actif	Type d'actif	Nombre actuellement possédé
1	Radio / radiocassette / chaine		5	Table		15	Voiture	
2	Téléviseur / vidéo		6	Chaise et pouf		16	Cyclomoteur / moto	
3	Téléphone fixe		7	Armoire		17	Tracteur	
4	Téléphone mobile		8	Lit		18	Charrette / calèche	
			9	Réfrigérateur		19	Bicyclette	
			10	Congélateur		20	Bateau / pirogue	
			11	Machine à laver		21	Autres véhicules	
			12	Micro-onde				
			13	Ventilateur				
			14	Climatiseur				

K11. Indiquez **qui prend** les **décisions** dans **votre ménage**.

N°		**Dans votre ménage**, qui **prend** les **décisions** concernant :
		Code K1
1	**L'éducation** des **garçons**	
2	**L'éducation** des **filles**	
3	**L'achat** des **vêtements**	
4	**L'achat** de la **nourriture** consommée par le ménage	
5	**L'achat** des **outils électroniques** (radio, TV, …)	
6	Les **programmes** sur la **télé/radio** qu'on regarde/écoute	
7	**L'achat** des **journaux/magazines**	
8	**L'achat** des **véhicules** (voiture, moto, bicyclette, …)	
9	**L'achat** des **téléphones (**mobiles**)**	
10	Dépenses pour **améliorer** la **maison**	
11	Des **visites** au **docteur**/poste de santé	
12	Le **nombre** des **enfants**	
13	Le **nombre** des **conjoints**	
14	**Limitation** des **naissances**	
15	**L'âge** quand vos **enfants** se **marient**	
16	Le **choix** de la **deuxième femme**	
17	Le **choix** des **conjoints** de vos **enfants**	
18	Le **travail** du **CM** dehors du ménage et la ferme	
19	Le **travail** du **conjoint** dehors du ménage et la ferme	
20	**Demande** de **crédit**	
21	Les **investissements** dans la **production agricole**	
22	Les **investissements** dans **l'élevage**	
23	Les **investissements** dans la **pêche**	
24	Les **investissements** dans le **business non-agricole**	
25	Des **visites** des **conjoints** aux amis / famille **dehors du village**	

CODE K1

1=Je décide seule (le mari)
2=Je décide ensemble avec la (les) conjointe(s) mais j'ai le dernier mot
3=Je décide ensemble avec la (les) conjointe(s) mais mon opinion est plus importante
4=Je décide ensemble avec la (les) conjointe(s) – avec consensus
5=Je décide ensemble avec la (les) conjointe(s) mais l'opinion de la (des) conjointe(s) est plus importante
6=Je décide ensemble avec la (les) conjointe(s) mais la (les) conjointe(s) a (ont) le dernier mot
7=Je ne décide pas, la (les) conjointe(s) décide(nt) seule

K12. Indiquez votre **degré d'accord** avec les **propositions** suivantes :

N°		Code K2
1	C'est **important** que les **enfants** peuvent aller à **l'école primaire.**	
2	C'est **important** que les **enfants** peuvent aller à **l'école secondaire**.	
3	Après la nourriture, l'**éducation** des enfants est la plus grande **priorité** du ménage.	
4	C'est plus **utile** que les **enfants travaillent** dans le ménage ou aux champs qu'ils vont à l'école.	
5	Vu leur position dans la société, des **garçons** doivent être **mieux scolarisés** que des **filles**.	
6	Un **enfant** qui est allé à **l'école** aura une **meilleure vie** qu'un enfant qui n'est **pas** allé à l'école.	

CODE K2

1=Tout à fait d'accord
2=D'accord
3=Plus ou moins d'accord
4=Neutre
5=Plus ou moins pas d'accord
6=Pas d'accord
7=Pas du tout d'accord

K13. Pendant les **dernières années**, comment est-ce que les **conditions** de **vie** ont **changés** pour votre **ménage** ?

0 Améliorées beaucoup [1] 0 Améliorées [2] 0 Pas changées [3] 0 Détériorées [4] 0 Détériorées beaucoup [5]

K14. En façon général, êtes-vous **heureux**? 0 très heureux [1] 0 heureux [2] 0 pas heureux / pas malheureux [3]
0 malheureux [4] 0 très malheureux [5]

Section L: Information et media

Qui du ménage est **responsable** pour **faire** le **ménage** ?

Demandez à **parler** avec cette femme et notez le nom et le code ID de la répondante à la page 1. De cette section jusqu'au fin de l'enquête, les questions sont adressés à cette femme.

L1. Pouvez-vous décrire pour chaque **membre** du ménage qui a **au moins 18 ans** son utilisation des **sources de media** et **les organisations** dont ils sont membres.
Les personnes dans le tableau sont les mêmes personnes comme au tableau C1 (chaque adulte dans le ménage) !

Membre du ménage	Regarde-t-il (elle) le journal sur la télévision ?	Ecoute-t-il (elle) le journal sur la radio ?	Utilise-t-il (elle) sa **propre** téléphone (fixe ou mobilier) ?	Lit-t-il (elle) des journaux ?	Utilise-t-il (elle) l'internet ?	Visite-t-il (elle) son profil aux médias sociaux ?	Est-il (elle) membre d'une organisation ?	**Si non :** la raison principale pour ne pas être membre	**Si oui :** quel type d'organisation + depuis quand **(plusieurs réponses possibles)**	A-t-il (elle) une **fonction politique**, **administrative** ou **publique** dans le village ou dans la CR ?	**Si oui :** depuis quand ?
Code ID	Code L1	Code L1	Code L1	Code L1	Code L1	Code L1	1=Oui 2=Non	Code L2	Code L3 + année	1 =Oui 2=Non	année
1											
2											
3											
4											
5											
6											
7											
8											
9											
10											
11											
12											
13											
14											
15											
16											
17											
18											
19											
20											

CODE L1

1=Une ou plusieurs fois par jour
2=Une ou plusieurs fois par semaine
3=Une ou plusieurs fois par mois
4=Une ou plusieurs fois par année
5=Jamais

CODE L2

0=Il n'y a pas d'organisations dans le village
1=Manque de temps
2=Manque d'argent pour contribuer à l'organisation
3=L'investissement de temps est trop pour ce qu'on reçoit
4=L'investissement financier est trop pour ce qu'on reçoit
5=La distance entre le ménage et l'endroit où l'organisation se réunit est trop grande
6=Pas d'intéresse
7=Pas de permission du conjoint
8=Autre (spécifiez)

CODE L3

1=GIE
2=Organisation villageoise
3=Organisation des paysans/paysannes
4=Groupement des femmes
5=Groupement de crédit et d'épargne
6=Organisation des sports
7=Organisation de jeunesse
8=Organisation religieuse
9=Autre (spécifiez)

Section M: Sécurité alimentaire et santé

M1. Pouvez-vous répondre des questions suivantes concernant vos **repas** et la **nourriture** que vous et votre **ménage** avez consommé pendant les **12 derniers mois** .

			**Si oui :** en quels mois? **(Indiquez avec un x)**											
No	**Pendant les 12 derniers mois**,	1=Oui 2=Non	Jan	Fév	Mar	Avril	Mai	Juin	Jui	Aout	Sep	Oct	Nov	Déc
1	existaient-ils des mois pendant lesquels vous n'aviez **pas assez de nourriture** pour satisfaire les besoins de votre famille?													
2	il y avait des jours dont le ménage n'a pas pu prendre **3 repas** ?													
3	il y avait des jours dont le ménage n'a pas pu prendre **2 repas**?													
4	il y avait des jours dont le ménage n'a pas pu prendre **aucun repas** ?													

M2. Pouvez-vous répondre des questions suivantes concernant vos **repas** et la **nourriture** que vous et votre **ménage** avez consommé pendant les **4 dernières semaines**.

			**Si oui :** avec quelle fréquence ? **(Indiquez avec un x)**		
No	**Ces quatre dernières semaines**,	1=Oui 2=Non	Rarement (une ou deux fois)	Parfois (trois à 10 fois)	Souvent (plus de 10 fois)
1	étiez-vous préoccupé que votre ménage n'avait **pas assez de nourriture** ?				
2	est-ce que vous-même ou un membre de votre ménage n'a pas pu manger les **types de nourriture** que vous **préférez** à cause d'un **manque de ressources** ?				
3	est-ce que vous-même ou un membre de votre ménage a mangé une **variété limitée d'aliments** parce que les ressources n'étaient pas suffisantes ?				
4	est-ce que vous-même ou un membre de votre ménage a mangé **une nourriture que vous ne souhaitiez pas manger** à cause du manque de ressources pour obtenir d'autres types de nourriture ?				
5	est-ce que vous-même ou un membre de votre ménage a mangé **un repas plus petit** que vous n'auriez souhaité parce qu'il n'y avait pas assez à manger ?				
6	est-ce que vous-même ou un membre de votre ménage a mangé **moins de repas par jour** parce qu'il n'y avait pas assez de nourriture ?				
7	est-il arrivé que le ménage soit **sans nourriture** du tout parce qu'il n'y avait pas de ressources pour en acheter ?				
8	est-ce que vous-même ou un membre de votre ménage est **allé au lit en ayant faim** parce qu'il n'y avait pas assez de nourriture ?				
9	est-ce que vous-même ou un membre de votre ménage a passé toute une **journée sans manger** parce qu'il n'y avait pas assez de nourriture ?				

M3. Combien **d'enfants** sont nés vivants mais sont **morts avant l'âge d'une année** dans votre ménage pendant les **20 dernières années**? ____ **enfants**
Qui étaient leurs mères ? **Notez le Code ID.**

M4. Combien **d'enfants** sont **morts entre 1 et 5 ans** dans votre ménage pendant les **20 dernières années**? ____ **enfants**
Qui étaient leurs mères ? **Notez le Code ID.**

M5. Vous avez déjà **visité** une **case/poste de santé** dans votre vie? 0 Oui [1] 0 Non [2]
Si non : Pourquoi est-ce que vous n'y avez pas allé encore ? 0 Pas nécessaire [1] 0 Trop loin [2] 0 Trop chère [3]
0 Pas de permission du CM [4] 0 Préfère la médecine traditionnelle [5] 0 Manque d'argent [6] 0 Autre (spécifiez) [7] ______________________

M6. Indiquez votre **degré d'accord** avec les **propositions** suivantes :

No.		Code M1
1	C'est **important** que les **enfants** peuvent aller à **l'école primaire.**	
2	C'est **important** que les **enfants** peuvent aller à **l'école secondaire**.	
3	Après la nourriture, l'**éducation** des enfants est la plus grande **priorité** du ménage.	
4	C'est plus **utile** que les **enfants travaillent** dans le ménage ou aux champs qu'ils vont à l'école.	
5	Vu leur position dans la société, des **garçons** doivent être **mieux scolarisés** que des **filles**.	
6	Un **enfant** qui est allé à **l'école** aura une **meilleure vie** qu'un enfant qui n'est **pas** allé à l'école.	

CODE M1
1=Tout à fait d'accord
2=D'accord
3=Plus ou moins d'accord
4=Neutre
5=Plus ou moins pas d'accord
6=Pas d'accord
7=Pas du tout d'accord

Section N: Prendre des décisions

Indiquez par sujet comme les **décisions** sont **pris** dans le **ménage** selon la femme. **Attention**, dans la **première part du tableau**, demandez **comment la femme voudrait prendre des décisions.** Ca contraste avec la **deuxième part du tableau** où vous demandez **comment les décisions sont pris en réalité**.

N1. Indiquez premier qui prendrait les décisions selon vous et puis, comment les décisions sont pris en réalité dans votre ménage.

		**Selon vous**, qui **prendrait** les **décisions** concernant :	**Dans votre ménage**, qui **prend** les **décisions** concernant :
No.		Code N1	Code N1
1	**L'éducation** des **garçons**		
2	**L'éducation** des **filles**		
3	**L'achat** des **vêtements**		
4	**L'achat** de la **nourriture** consommée par le ménage		
5	**L'achat** des **outils électroniques** (radio, TV, …)		
6	Les **programmes** sur la **télé/radio** qu'on regarde/écoute		
7	**L'achat** des **journaux/magazines**		
8	**L'achat** des **véhicules** (voiture, moto, bicyclette, …)		
9	**L'achat** des **téléphones (**mobiles**)**		
10	Dépenses pour **améliorer** la **maison**		
11	Des **visites** au **docteur**/poste de santé		
12	Le **nombre** des **enfants**		
13	Le **nombre** des **conjoints**		
14	**Limitation** des **naissances**		
15	**L'âge** quand vos **enfants** se **marient**		
16	Le **choix** de la **deuxième femme**		
17	Le **choix** des **conjoints** de vos **enfants**		
18	Le **travail** du **CM** dehors du ménage et la ferme		
19	Le **travail** du **conjoint** dehors du ménage et la ferme		
20	**Demande** de **crédit**		
21	Les **investissements** dans la **production agricole**		
22	Les **investissements** dans **l'élevage**		
23	Les **investissements** dans la **pêche**		
24	Les **investissements** dans le **business non-agricole**		
25	Des **visites** des **conjoints** aux amis / famille **dehors du village**		

CODE N1

1=Le mari décide seule
2=Je décide ensemble avec la (les) conjointe(s) mais il a le dernier mot
3=Je décide ensemble avec la (les) conjointe(s) mais son opinion est plus importante
4=Je décide ensemble avec la (les) conjointe(s) – avec consensus
5=Je décide ensemble avec la (les) conjointe(s) mais l'opinion de la (des) conjointe(s) est plus importante
6=Je décide ensemble avec la (les) conjointe(s) mais la (les) conjointe(s) a (ont) le dernier mot
7=Le mari décide pas, la (les) conjointe(s) décide(nt) seule

N2. Pouvez-vous sortir sans la permission de votre mari? 0 Oui [1] 0 Non [2] 0 Dépend d'où je sors [3]

N3. Pendant les **dernières années**, comment est-ce que les **conditions** de **vie** ont **changés** pour votre **ménage** ?
0 Améliorées beaucoup [1] 0 Améliorées [2] 0 Pas changées [3] 0 Détériorées [4] 0 Détériorées beaucoup [5]

N4. En façon général, êtes-vous **heureux**? 0 très heureux [1] 0 heureux [2] 0 pas heureux / pas malheureux [3] 0 malheureux [4] 0 très malheureux [5]

N5. **Pour l'enquêteur :** indiquez si le mari était présent dans cette part de l'enquête 0 Oui [1] 0 Non [2]

Fin de l'enquête :
Merci beaucoup pour votre coopération et votre temps !
