# Supplementary material for: Female Employment Reduces Fertility in Rural Senegal
Source: PLoS One. 2015 Mar 27;10(3):e0122086. doi: 10.1371/journal.pone.0122086 (PMC4376695; doi:10.1371/journal.pone.0122086)
Supplement: S1 Table — Source: own calculations from survey data. Comparisons are made between households with female wage employment and households without female wage employment using t-tests. Standard errors are reported in parentheses. Significant differences are indicated with * p<0.1, ** p<0.05 or *** p<0.01. (PDF) [file pone.0122086.s004.pdf]

Table S 1. Means comparison of income indicators for households with and without female employment
***Source*: own calculations from survey data.**

	Total	Households without female employment	Households with female employment	
Household total income (FCFA)	2,682,325	2,551,522	3,046,989	*
	(140,699)	(168,253)	(251,295)	
Household agricultural income (FCFA)	1,039,152	1,179,789	647,072	**
	(117,016)	(146,591)	(167,743)	
Household self-employment income (FCFA)	644,591	635,869	668,909	
	(49,913)	(58,634)	(95,336)	
Household non-labour income (FCFA)	350,183	367,061	303,129	
	(37,992)	(46,895)	(60,227)	
Male wage income (FCFA)	460,332	367,580	718,912	***
	(57,651)	(66,416)	(113,182)	
Female wage income (FCFA)	187,168	0	708,968	***
	(20,004)	(0)	(54,313)	
Number of observations	500	368	132	

Comparisons are made between households with female wage employment and households without female wage employment using *t*-tests. Standard errors are reported in parentheses. Significant differences are indicated with * $p<0.1$, ** $p<0.05$ or *** $p<0.01$.
