# Supplementary material for: Female Employment Reduces Fertility in Rural Senegal
Source: PLoS One. 2015 Mar 27;10(3):e0122086. doi: 10.1371/journal.pone.0122086 (PMC4376695; doi:10.1371/journal.pone.0122086)
Supplement: S2 Table — Source: own calculations from survey data. Comparisons are made between wage employed women and non-wage employed women using t-tests. Significant differences are indicated with * p<0.1, ** p<0.05 or *** p<0.01. a One tropical livestock unit (TLU) equals 1 cow/horse, 0.8 donkey, and 0.2 sheep/goat. b The Multidimensional Poverty Index (MPI) is calculated according to the guidelines by the United Nations Development Program [28]. (PDF) [file pone.0122086.s005.pdf]

Table S 2. Means comparison of individual, household and village characteristics for employed and non-employed women. ***Source*****: own calculations from survey data.**

Characteristics	Total		Not wage employed		Wage employed		
	Mean	St. Dev.	Mean	St. Dev.	Mean	St. Dev.	
Individual characteristics							
Age	26.79	6.51	26.39	6.51	28.57	6.21	***
Literacy	44.83%		42.98%		52.97%		***
Single	40.22%		38.55%		47.57%		**
Wife of HH head	21.46%		22.54%		16.76%		**
Daughter or granddaughter	43.03%		40.15%		55.68%		***
Household characteristics							
Religion (1 = Christian)	3.01%		2.83%		3.78%		
Ethnicity (1 = Wolof)	48.75%		48.65%		49.19%		
Ethnicity (1 = Pular)	36.61%		37.68%		31.89%		*
Gender HH head (1 = female)	11.63%		11.21%		13.51%		
Age HH head	57.62	13.63	57.27	13.75	59.15	13.01	**
Literacy HH head	28.89%		26.48%		39.46%		***
Land owned (ha)	2.91	7.14	3.02	7.08	2.43	7.39	
Livestock units [a]	10.14	43.75	11.55	48.13	3.95	10.08	**
Poor Household (MPI>33) [b]	46.74%		47.41%		43.78%		
Village characteristics							
Female organization in village	45.14%		41.26%		62.16%		***
Distance to road (km)	2.34	3.14	2.55	3.23	1.43	2.58	***
Multiple ethnicities in village	66.20%		65.64%		68.65%		
Number of observations	997		812		185		

Comparisons are made between wage employed women and non-wage employed women using *t*-tests. Significant differences are indicated with * p<0.1, ** p<0.05 or *** p<0.01.

[a] One tropical livestock unit (TLU) equals 1 cow/horse, 0.8 donkey, and 0.2 sheep/goat.

[b] The Multidimensional Poverty Index (MPI) is calculated according to the guidelines by the United Nations Development Programme [28].
