# Supplementary material for: Female Employment Reduces Fertility in Rural Senegal
Source: PLoS One. 2015 Mar 27;10(3):e0122086. doi: 10.1371/journal.pone.0122086 (PMC4376695; doi:10.1371/journal.pone.0122086)
Supplement: S3 Table — Source: own estimations from survey data. The first column reports the simple DD regression Y i = β 0 +β 1 T i +β 2 t+β 3 T i t+ε i where Y is fertility, T is female employment and t is the year. The second column reports the DD regression when additional observable characteristics are taken into account: Y i = β 0 +β 1 T i +β 2 t+β 3 T i t+β k X ik +ε i, where X is a vector of individual and household characteristics observed in 2005 and 2013. The third column reports the DD regression after matching treated observations with untreated observations based on an estimated propensity score and using Kernel matching, and controlling for X. The propensity score is estimated as the probability of employment conditional on X. Standard errors are reported in parentheses. Significant effects are indicated with * p<0.1, ** p<0.05 or *** p<0.01. (PDF) [file pone.0122086.s006.pdf]

Table S 3. Regression results of difference-in-differences estimations on fertility. *Source*: own estimations from survey data.

	DD		DD with covariates		DD with PSM	
Year	1.283	***	0.581	***	0.679	***
	(0.069)		(0.057)		(0.073)	
Wage employment	-0.011		-0.113	**	-0.034	
	(0.033)		(0.052)		(0.050)	
Employed * Year	-0.332	**	-0.291	**	-0.320	**
	(0.139)		(0.122)		(0.130)	
Age			0.070	***	0.057	***
			(0.006)		(0.006)	
Literacy			-0.249	***	-0.230	***
			(0.053)		(0.067)	
Single			-0.503	***	-0.682	***
			(0.063)		(0.071)	
Religion (1=christian)			-0.291	**	-0.490	***
			(0.138)		(0.161)	
Ethnicity (1=Wolof)			-0.100		-0.178	*
			(0.080)		(0.100)	
Ethnicity (1=Pular)			0.029		-0.036	
			(0.085)		(0.110)	
Land owned (ha)			-0.001		0.007	
			(0.003)		(0.008)	
Distance to concrete road (km)			0.013		0.017	
			(0.010)		(0.015)	
Constant	0.119	***	-0.697	***	-0.348	**
	(0.016)		(0.152)		(0.169)	
Number of observations	1994		1994		1990	
R-squared	0.174		0.378		0.379	

The first column reports the simple DD regression $Y_i = \beta_0 + \beta_1 T_i + \beta_2 t + \beta_3 T_i t + \varepsilon_i$ where *Y* is fertility, *T* is female employment and *t* is the year. The second column reports the DD regression when additional observable characteristics are taken into account: $Y_i = \beta_0 + \beta_1 T_i + \beta_2 t + \beta_3 T_i t + \beta_k X_{ik} + \varepsilon_i$, where *X* is a vector of individual and household characteristics observed in 2005 and 2013. The third column reports the DD regression after matching treated observations with untreated observations based on an estimated propensity score and using Kernel matching, and controlling for *X*. The propensity score is estimated as the probability of employment conditional on *X*. Standard errors are reported in parentheses. Significant effects are indicated with * p<0.1, ** p<0.05 or *** p<0.01.
