# Supplementary material for: Female Employment Reduces Fertility in Rural Senegal
Source: PLoS One. 2015 Mar 27;10(3):e0122086. doi: 10.1371/journal.pone.0122086 (PMC4376695; doi:10.1371/journal.pone.0122086)
Supplement: S4 Table — Source: own estimations from survey data. Balancing properties are tested so that pretreatment characteristics of treated and control units do not differ significantly after matching. Significant differences are indicated with * p<0.1, ** p<0.05 or *** p<0.01. (PDF) [file pone.0122086.s007.pdf]

Table S 4. Balancing properties of variables in treated and control groups for kernel matching on propensity scores. ***Source*****: own estimations from survey data.**

		Mean treated units	Mean control units	% Bias between treated and controls	% Reduction in bias	t-test: Mean(control)= Mean(treatment)
Age	Unmatched	20.57	18.39	34.3		4.15***
	Matched	20.46	20.36	1.6	95.3	0.15
Single	Unmatched	0.69	0.68	2.0		0.24
	Matched	0.68	0.67	2.5	-25.7	0.23
Literacy	Unmatched	0.53	0.43	20.1		2.47**
	Matched	0.53	0.52	2.6	87.2	0.24
Ethnicity (1=Pular)	Unmatched	0.32	0.38	-12.2		-1.48
	Matched	0.32	0.32	-0.5	96.1	-0.05
Ethnicity (1=Wolof)	Unmatched	0.49	0.49	1.1		0.13
	Matched	0.50	0.49	1.9	-75.4	0.18
Religion (1=christian)	Unmatched	0.04	0.03	5.3		0.68
	Matched	0.04	0.04	-0.3	93.5	-0.03
Land owned (ha)	Unmatched	1.89	2.83	-15.9		-1.75*
	Matched	1.88	1.91	-0.5	96.8	-0.06
Distance to concrete road (km)	Unmatched	1.43	2.55	-38.2		-4.39***
	Matched	1.45	1.49	-1.6	95.8	-0.18

Balancing properties are tested so that pretreatment characteristics of treated and control units do not differ significantly after matching. Significant differences are indicated with * p<0.1, ** p<0.05 or *** p<0.01.
