# Supplementary material for: Female Employment Reduces Fertility in Rural Senegal
Source: PLoS One. 2015 Mar 27;10(3):e0122086. doi: 10.1371/journal.pone.0122086 (PMC4376695; doi:10.1371/journal.pone.0122086)
Supplement: S5 Table — Source: own estimations from survey data. The first column reports the average marginal effects of the fertility determinants from a cross-sectional Poisson regression, controlling for individual, household and village characteristics. The second column reports the average marginal effects of the fertility determinants from a cross-sectional Poisson regression, controlling for individual and household characteristics and village fixed effects. The last column reports the average marginal effects of the fertility determinants from a 2SRI model. In the first stage, the distance to the nearest horticultural export company is used as an instrument for female employment. Standard errors are reported in parentheses. Significant effects are indicated with * p<0.1, ** p<0.05 or *** p<0.01. (PDF) [file pone.0122086.s008.pdf]

Table S 5. Regression results of Poisson estimations on fertility. ***Source*****: own estimations from survey data.**

	Poisson		Village FE		2SRI	
Wage employment	-0.256	**	-0.215	*	-0.289	
	(0.109)		(0.125)		(0.557)	
Age	0.471	***	0.469	***	0.473	***
	(0.075)		(0.078)		(0.090)	
Age²	-0.007	***	-0.007	***	-0.007	***
	(0.001)		(0.001)		(0.001)	
Literacy	-0.248	**	-0.243	**	-0.248	**
	(0.101)		(0.107)		(0.106)	
Single	-1.553	***	-1.526	***	-1.551	***
	(0.293)		(0.281)		(0.307)	
Number of children in 2005	0.207	***	0.179	***	0.208	***
	(0.056)		(0.066)		(0.066)	
Wife of HH head	0.981	***	1.023	***	0.979	***
	(0.135)		(0.139)		(0.139)	
(Grand)daughter of HH head	-0.889	***	-0.877	***	-0.886	***
	(0.196)		(0.194)		(0.208)	
Religion (1=christian)	-0.635	**	-0.652	*	-0.640	*
	(0.293)		(0.351)		(0.325)	
Ethnicity (1=Wolof)	-0.087		-0.055		-0.092	
	(0.124)		(0.240)		(0.136)	
Ethnicity (1=Pular)	0.013		-0.193		-0.017	
	(0.128)		(0.245)		(0.130)	
Gender HH head (1=female)	0.357	***	0.369	**	0.358	***
	(0.129)		(0.154)		(0.133)	
Age HH head	0.012	***	0.011	**	0.012	***
	(0.004)		(0.005)		(0.004)	
Literacy HH head	-0.126		-0.117		-0.124	
	(0.094)		(0.096)		(0.107)	
Land owned (ha)	-0.004		-0.008		-0.004	
	(0.005)		(0.007)		(0.007)	
Livestock units	0.000		0.000		-0.000	
	(0.001)		(0.001)		(0.001)	
Poor household (MPI>33)	0.184	**	0.191	**	0.185	**
	(0.082)		(0.089)		(0.084)	
Female organisation in village	-0.011				-0.007	
	(0.099)				(0.112)	
Multiple ethnicities in village	0.058				0.056	
	(0.116)				(0.121)	
Distance to concrete road (km)	0.004				0.003	
	(0.016)				(0.016)	
Residuals					0.035	
					(0.567)	
Number of observations	997		997		997	
Log Likelihood	-1150.86		-1130.41		-1150.86	
Chi²	1197.69		1321.49		1059.68	
Prob > Chi²	0		0		0	
Pseudo R²	0.39		0.41		0.39	

The first column reports the average marginal effects of the fertility determinants from a cross-sectional Poisson regression, controlling for individual, household and village characteristics. The second column reports the average marginal effects of the fertility determinants from a cross-sectional Poisson regression, controlling for individual and household characteristics and village fixed effects. The last column reports the average marginal effects of the fertility determinants from a 2SRI model. In the first stage, the distance to the nearest horticultural export company is used as an instrument for female employment. Standard errors are reported in parentheses. Significant effects are indicated with * $p<0.1$, ** $p<0.05$ or *** $p<0.01$.
