# Supplementary material for: Female Employment Reduces Fertility in Rural Senegal
Source: PLoS One. 2015 Mar 27;10(3):e0122086. doi: 10.1371/journal.pone.0122086 (PMC4376695; doi:10.1371/journal.pone.0122086)
Supplement: S6 Table — Source: own estimations from survey data. The table reports the Ordinary Least Square coefficient estimates of the first stage of the Two-stage Residual Inclusion. Probability of being employed is regressed on individual, household and village characteristics, and the distance to the nearest horticultural export company is used as instrument. Standard errors are reported in parentheses. Significant effects are indicated with * p<0.1, ** p<0.05 or *** p<0.01. (PDF) [file pone.0122086.s009.pdf]

Table S 6. 2SRI estimation first stage regression results on the likelihood of employment. *Source*: own estimations from survey data.

	Coefficient	Standard error	
Age	0.052	(0.018)	***
Age²	-0.001	(0.000)	**
Literacy	0.003	(0.027)	
Single	0.070	(0.033)	**
Number of children in 2005	0.016	(0.033)	
Wife of HH head	-0.050	(0.043)	
(Grand)daughter of HH head	0.087	(0.031)	***
Religion (1=Christian)	-0.070	(0.077)	
Ethnicity (1=Wolof)	-0.082	(0.043)	*
Ethnicity (1=Pular)	-0.040	(0.046)	
Gender HH head (1=female)	0.057	(0.038)	
Age HH head	-0.001	(0.001)	
Literacy HH head	0.044	(0.030)	
Land owned (ha)	0.001	(0.002)	
Livestock units	0.000	(0.000)	
Poor household (MPI>33)	0.022	(0.025)	
Female organization in village	0.069	(0.030)	**
Multiple ethnicities in village	-0.117	(0.032)	***
Distance to concrete road (km)	-0.010	(0.005)	**
Distance to company (km)	-0.004	(0.001)	***
Constant	-0.520	(0.262)	**

The table reports the Ordinary Least Square coefficient estimates of the first stage of the Two-stage Residual Inclusion. Probability of being employed is regressed on individual, household and village characteristics, and the distance to the nearest horticultural export company is used as instrument. Standard errors are reported in parentheses. Significant effects are indicated with * p<0.1, ** p<0.05 or *** p<0.01.
